# Daily and Half-yearly Associations between Boundary Diffusion and Parent-adolescent Relationship Quality after Divorce

**DOI:** 10.1007/s10964-024-02064-w

**Published:** 2024-09-09

**Authors:** Rianne van Dijk, Stefanos Mastrotheodoros, Inge E. van der Valk, Susan Branje, Maja Deković

**Affiliations:** 1 Department of Clinical Child & Family Studies, Utrecht University, Utrecht, The Netherlands; 2 Department of Youth & Family, Utrecht University, Utrecht, The Netherlands; 3 Department of Psychology, University of Crete, Rethymno, Greece

**Keywords:** Parent-adolescent relationship quality, Boundary diffusion, Divorce, Adolescence, Daily and long-term associations

## Abstract

Boundary diffusion is a particular risk after divorce and has been associated with adolescents’ adjustment problems. Yet, its potential impact on parent-adolescent relationship quality is less straightforward, as previous findings support both an *alienation* and *conflict perspective*. Therefore these associations (daily and half-yearly) were examined in recently divorced families, addressing both within-dyad changes and between-dyad differences. Data were collected among a sample of *N* = 133 (pre)adolescents (*M*_*age*_ = 11.76; 51.5% boys) from 76 divorced families, using a measurement burst design: Every six months, 14 consecutive days of daily diaries were collected, for 5 waves. Between dyads, adolescents who experienced more boundary diffusion than others, also reported more conflict with both their parents. Within dyads, when adolescents experienced more boundary diffusion than usual by one of their parents (actor), warmth decreased and conflict increased between this parent and the adolescent, that same and the following day. Adolescents also engaged in more conflict with the other parent that day. These findings mostly supported the conflict perspective: Post-divorce boundary diffusion appears to be a general risk factor for parent-adolescent conflict with both parents, and from day-to-day boundary diffusion was linked to a deteriorated parent-adolescent relationship quality, especially with the parent that triangulated or parentified them. There were no significant long-term associations, nor did any moderator (age, gender, living arrangement) explain heterogeneity in effects.

## Introduction

Parental divorce or separation is a major life event for all family members involved, including the children. Compared to their peers from intact families, they are at risk for cross-generational boundary diffusion, or the blurring of hierarchical boundaries that define developmentally suitable family roles (Kerig, 2016; Perrin et al., 2013), which is thought to put children in a confusing and distressing position (Minuchin, 1974). Although boundary diffusion has indeed been related to poor child adjustment in previous studies (e.g., Baker & Brassard, 2013; Perrin et al., 2013), its potential impact on the qualitative aspects of the parent-child relationship – such as warmth and conflict – is less well understood. A better understanding of the association between post-divorce boundary diffusion and parent-child relationship quality is warranted, as the latter is generally rated lower in divorced than in intact families (Afifi & Schrodt, 2003; Mustonen et al., 2011), and parents tend to be less available and responsive to their children’s needs during the first few years after divorce (Sutherland et al., 2012; Wallerstein et al., 2013). Given the developmental changes in parent-adolescent relationship quality including a normative decrease in warmth (Mastrotheodoros et al., 2019a) and an increase in conflict (Mastrotheodoros et al., 2020), it is particularly important to examine whether these changes are exacerbated by boundary diffusion during adolescence. The current study examined the associations between post-divorce boundary diffusion and parent-adolescent relationship quality – on a within and between level – with both parents, and on a daily and half-yearly timescale. By discerning the within- and between-dyad associations, it is possible to differentiate between changes that occur within parent-adolescent dyads as is postulated in family theories on the one hand, and stable differences between dyads to identify those most at risk for post-divorce adversity on the other hand (Keijsers & Van Roekel, 2018). It was also assessed whether differences in within-dyad associations may depend on adolescents’ age, gender, and their post-divorce living arrangement.

### Boundary Diffusion and Parent-adolescent Relationship Quality

Although various conceptualizations of boundary diffusion exist in the literature (e.g., Kerig, 2005), parental triangulation and emotional parentification are the most commonly described in the context of divorce, and are thought to co-occur and sustain each other (e.g., Garber, 2011; Perrin et al., 2013). Triangulation refers to parents inappropriately involving a child into the interparental subsystem, for example in parental disputes (Baker & Eichler, 2014; Kerig & Swanson, 2010). This occurs by pressuring children to take sides, using them as a messenger or “go-between”, and badmouthing or disclosing negative information about the other parent to children (Buehler & Welsh, 2009; Kerig & Swanson, 2010). Emotional parentification refers to parents relying on their child for support to meet their own emotional needs, thereby compromising those of the child (Jankowski et al., 2013). Previous findings are inconsistent regarding the potential benefits or harms of boundary diffusion for children’s relationships with both parents, and can be explained by two opposing views, the *alienation perspective*^1^ and the *conflict perspective*.

The *alienation perspective* assumes that boundary diffusion fosters a close relationship, or alliance, between the parent who uses these behaviors (actor parent) and their children, while distancing them from the targeted “other” parent (Rowen & Emery, 2018). Being needed by their parent would increase children’s sense of responsibility in caring for others and thus their feelings of closeness (Borchet et al., 2020; Hooper, 2007), while compromising their bond with the other parent due to copying the actor parent’s negative evaluation of the ex-partner. Research indeed shows that in divorced families where parents denigrated the other parent more, adolescents generally felt closer to the actor parent (often mother; Afifi & McManus, 2010), and less close to the other parent (often father; Kenyon & Koerner, 2008) compared to families with low levels of boundary diffusion. The latter was only found for boys with lower emotional autonomy and adolescents who strongly identified with and reacted to their mothers’ emotions. Likewise, adolescents from mostly intact families who experienced more parentification from one of their parents, reported more closeness to that parent, compared to those who reported less emotional parentification (Lichtwarck-Aschoff et al., 2012; O’Mara & Schrodt, 2017). These findings underscore the importance of examining boundary diffusion during adolescence particularly, as parent-adolescent relationships transform during this developmental period (Branje et al., 2021).

According to the *conflict perspective*, boundary diffusion is considered a form of conflict between parents that partly is continued through their children (Grych et al., 2004; Rowen & Emery, 2018). Because interparental conflict is detrimental for the parent-child relationship after divorce (Van Dijk et al., 2020), boundary diffusion would be associated with worse parent-adolescent relationship quality with both parents. A parent’s negative evaluation of the other parent could also cause children to feel rejected by the actor parent (Baker & Ben-Ami, 2011), as they are inherently connected to both of their parents, relationally and often genetically. Adolescents from divorced families indeed tend to report lower self-esteem when they experience more parental triangulation (Van Dijk et al., 2022). Congruent with the conflict perspective, young adults from intact and divorced families that perceived higher levels of parental denigration, also reported feeling more distant from both their parents (Rowen & Emery, 2018, 2019). In intact families, parental triangulation was associated with parent-adolescent conflict concurrently as well as six months later (Fosco & Grych, 2010), and adolescents who had more emotionally reliant parents, reported less maternal and paternal warmth than adolescents who experienced low levels of emotional parentification (Peris et al., 2008).

The current study aimed to extend previous work in several ways. First, associations were examined between boundary diffusion and parent-adolescent relationship quality in recently divorced families, in which there is a higher risk for boundary diffusion and for poor parent-adolescent relationship quality, when compared to intact families (Peris & Emery, 2005; Perrin et al., 2013). Second, these associations were tested for parent-adolescent relationship quality with both the actor and the other parent, and on a daily and half-yearly timescale. Because parent-adolescent dynamics unfold and fluctuate on a daily basis (Keijsers & Van Roekel, 2018), and these daily interactions are considered to drive long-term development (Repetti et al., 2015), examining daily as well as long-term associations in parent-adolescent dyads can benefit the understanding of their relationships after divorce. Perhaps one incident of boundary diffusion on a specific day will not affect parent-adolescent warmth or conflict that same day or the consecutive day, but cumulative instances of boundary diffusion might affect relationship quality over a longer time scale. Contrarily, one incident of boundary diffusion on a specific day might directly affect parent-adolescent warmth and conflict, but this effect might dissipate soon, such that accumulating boundary diffusion does not affect long-term relationship quality. Current knowledge on daily dynamics is lacking, whereas in interventions aimed at preventing negative effects of divorce, the emphasis is precisely on changing such daily interactions (e.g., Dillman Taylor et al., 2011; Zhou et al., 2008). Third, most research has examined whether dyads with more boundary diffusion also show a lower or higher relationship quality than dyads with less boundary diffusion, which reflects a *between-dyad* association. The potential changes in boundary diffusion and their effects on relationship quality, however, occur *within-dyads*. Previous work showed that between-dyad associations do not necessarily translate into within-dyad effects (e.g., Mastrotheodoros et al., 2019b). Examining within-dyad effects also aligns better with intervention efforts aimed at parent-adolescent relationships after divorce (e.g., Vélez et al., 2011), as it is presumed that the impact of boundary diffusion – as well as trying to alter such family dynamics – occurs within that specific dyad.

### Potential Heterogeneity

It is to assume that dyads differ in how boundary diffusion associates with parent-adolescent relationship quality, resulting in differences between dyads in their within-dyad dynamics (Boele et al., 2020; Keijsers & Van Roekel, 2018). Whereas the conflict perspective may apply to some dyads after divorce, alienation could be apparent in other parent-adolescent dyads. This potential heterogeneity possibly depends on adolescents’ characteristics, such as age and gender, or their post-divorce living arrangements.

#### Adolescents’ age

Older adolescents might be less prone to bond with the actor parent as compared to younger adolescents. In light of cognitive maturation, younger adolescents potentially lack the social-cognitive processing skills – such as empathy and perspective taking – to experience boundary diffusion as something negative or harmful. They possibly feel as if they are favored by their parent or “being trusted with special privileges” instead (Peris & Emery, 2005), facilitating a better relationship with the actor parent and a worse relationship with the other parent. As social-affective processing gradually develop during adolescence (e.g., Crone & Dahl, 2012), older adolescents would therefore be more attuned to subtle interparental issues and their consequences (Van Eldik et al., 2020). Moreover, when parents rely on their children to meet their own personal needs, they could particularly hinder older adolescents in the development of individuation, autonomy, and independence (Grych et al., 2004; Zimmer-Gembeck & Collins, 2008). Boundary diffusion would then be more likely to result in a deteriorated parent-child relationship quality with both parents for older adolescents in contrast to younger adolescents.

#### Adolescents’ gender

Reasoning from gender socialization models, girls tend to place more importance on interpersonal relationships than boys (e.g., Davies & Lindsay, 2004), potentially making them more vulnerable to boundary diffusion. This implies that associations between boundary diffusion and parent-adolescent relationship quality would be stronger for girls. Research on the differentiating role of gender in the effect of boundary diffusion on (general) adjustment, however, is inconsistent. Whereas in some studies stronger effects of boundary diffusion were found for girls (Hetherington, 1999), other studies found a bigger impact on boys (Lichtwarck-Aschoff et al., 2012; Perrin et al., 2013), and yet in other studies there were no indications for gender differences (Perrin et al., 2013; Van Dijk et al., 2021). No studies seem to have examined gender differences in the impact of boundary diffusion on parent-adolescent relationship quality specifically.

#### Post-divorce living arrangement

Associations between parental denigration of the ex-spouse and parent-adolescent relationship quality seem to differ based on the custody arrangements. Whereas studies that found (partial) support for the alienation perspective were primarily based on samples where mothers had sole custody of the children (Afifi & McManus, 2010; Kenyon & Koerner, 2008), in studies supporting the conflict perspective, the number of shared residence arrangements was unknown (Rowen & Emery, 2018, 2019). Perhaps boundary diffusion can particularly result in a warm bond and few conflicts with the actor parent when there is only little contact with the other parent. More research is needed on the potential role of living arrangements in the link between boundary diffusion and parent-adolescent relationship quality. This is especially relevant because of the rising numbers of shared parenting arrangements after divorce (see Berman & Daneback, 2020; Nielsen, 2011 for reviews).

## Current Study

A better understanding of post-divorce boundary diffusion and parent-child relationship quality is needed, because the parent-adolescent relationship can be under pressure after divorce and boundary diffusion behaviors might explain relational changes. Since these dynamics are expected to occur within specific parent-adolescent dyads, this study disentangled within-dyad effects from between-dyad associations. To thoroughly examine the concurrent and longitudinal associations between boundary diffusion and parent-adolescent relationship quality, both warmth and conflict with the actor parent and the other parent were assessed on a daily and half-yearly timescale. Although there is support in the literature for both the alienation and conflict perspective, the differences in findings might be explained by the type of samples used. Prior findings congruent with the alienation perspective mostly included samples of complex divorces where primarily mothers had sole custody. In the current study interparental conflict was relatively low, almost all parents shared custody, and most adolescents spent a considerable amount of time with both parents. It was therefore expected that more support for the conflict perspective would be found, as opposed to the alienation perspective: Dyads with more boundary diffusion than other dyads would show lower levels of warmth and more conflict (between-dyad; Hypothesis 1a), and when an adolescent reports more boundary diffusion than the dyad’s own mean, they would experience less warmth and more conflict than usual with both parents (within-dyad; Hypothesis 1b). As between-dyad heterogeneity in the within-dyad associations was assumed (Hypothesis 2a), exploring factors that might explain this heterogeneity can help identify under which circumstances boundary diffusion could be more impactful. First, due to cognitive maturation and the emerging need for autonomy during adolescence, boundary diffusion may be more strongly linked to negative relationship quality with both parents for older adolescents compared to younger adolescents (Hypothesis 2b). Second, it was predicted to find stronger effects for girls than boys (Hypothesis 2c), because of gender socialization processes. Third, the alienation perspective was considered more likely when adolescents mostly live with one parent (typically mother; Hypothesis 2d).

## Methods

**Fig. 1:**
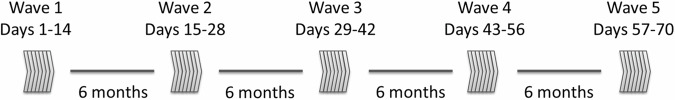
Visualization of the measurement burst design

The current study used data from the longitudinal research project “Family Dynamics after Divorce (FDD)”, which was conducted in the Netherlands (2016–2020). It consists of three annual measurement waves in which adolescents and their recently divorced parent(s) filled out questionnaires. Parallel to the annual waves, adolescents participated in a daily diary study with a measurement burst design. Every six months, 14 consecutive days of daily diaries were collected, for 5 waves (see Fig. 1). Because the current study focused on both short-term (daily) and long-term (half-yearly) associations, day-to-day effects were examined using the daily measures, and effects over 6 month periods using aggregations of daily measures. This way the measurements for the two different timescales were based on the same reports and could be more easily compared. This specific study was preregistered: https://osf.io/5y6av.

Recently divorced families were recruited through advertisements on websites for divorced parents, in school newsletters, and in waiting rooms of general practitioners, divorce mediators, and divorce counsellors. When families indicated their willingness to participate, they received further information about the study. Both custodial parents had to give active informed consent for the participation of their child(ren), and adolescents were asked for their written consent as well. In addition to the daily diary data used in the current study, adolescents and their parents filled out three annual surveys and they participated twice in parent-adolescent interaction tasks that were videotaped. Most of the data were gathered during home visits. The home visits were done by the principal investigator of the project, research assistants, and Graduate students of the Faculty of Social and Behavioral Sciences at Utrecht University.

Adolescents were asked to fill out 5 waves of online assessments on 14 consecutive days, with a 6-month interval. This resulted in maximally 70 daily assessments over the course of two years, in which they reported on daily family dynamics, parenting behaviors, and their mood at the end of each day. The adolescents started wave 1, 3, and 5 of the diary study after the annual home visit, during which the procedure was explained to them. Waves 2 and 4 were scheduled in between the home visits, when equivalent information on the procedure was sent by regular mail and parents were notified by e-mail. With both procedures, adolescents received information on how to download the App, what was expected of them, and when to fill out the daily assessments. They also received a calendar with 14 days they could cross-off with a pen or by using the stickers that were provided. Automatic notifications in the App were sent every evening, and researchers monitored the process of filling out the daily assessments. When adolescents omitted to fill out the daily assessments, reminders in the App were sent and the families were contacted through email or by phone. Each home visit, adolescents received a gift certificate of €10 for their participation.

### Participants

Of the original 135 who participated in the study, a total of *N* = 133 (pre)adolescent children took part in the diary study. They were part of 76 predominantly White, recently divorced families, as several adolescents belonged to the same family: In 9 families 3 children participated, in 39 families 2 children participated, and in 28 families 1 child participated. At T1, adolescents were between 7.8 and 16.9 years old (*M* = 11.76; *SD* = 2.31), and there were 68 boys (51.5%) and 65 girls. Almost all (94.7%) were born in the Netherlands, others in Morocco (0.8%), Surinam (0.8%), England (1.5%), Kenya (1.5%), and Slovakia (0.8%). A little over half (56.1%) of the adolescents went to primary school at T1, and 43.9% went to secondary school.

Parents had on average been living separately for 11.41 months (*SD* = 6.47) at T1, ranging from 1 week to 2.1 years. Mothers were between 29 and 55 years old (*M* = 43.46 years, *SD* = 5.64) and fathers were between 32 and 61 years old (*M* = 45.53, *SD* = 6.33). The highest attained education of mothers ranged from primary education (1.4%), high school (13.7%), vocational education (24.7%), and college education (41.1%) to university education (19.2%). Of the fathers, 12.5% finished high school, 17.9% vocational education, 39.3% college education, and 30.4% university education. In addition, 17.4% mothers and 1.9% fathers had a taxable monthly income below €1.250, 56.5% mothers and 32.1% fathers had an income between €1.250 and €3.750, and 26.1% mothers and 66.0% fathers earned over €3.750 per month. Both the average educational level and monthly income of mothers and fathers were higher than the Dutch national average (CBS, 2020, 2021)

### Daily Measures

Before answering the questions on family dynamics, adolescents had to answer whether they were staying at their mother’s (51.7%) or father’s house (27.4%), or whether they switched houses that specific day (20.9%). If they indicated that they were staying somewhere else (e.g., at camp, with friends or at their grandparents), that specific record was deleted from the data (*k* = 20). If they reported staying with one parent, adolescents were asked whether they have had contact with their other parent that specific day, either by phone, the internet, or through other means. When staying at their mother’s house, 41.2% of the time they reported having contact with their father that day. When they stayed with their father, 51.5% of the time they had contact with their mother that day. If adolescents indicated they had no contact with the non-residential parent that day, the questions on boundary diffusion and relationship quality were omitted for that parent. Missing data also occurred when participants failed to complete a daily assessment or an entire measurement burst. The mean number of days adolescents filled out the assessment ranged from 8.2 (W5) to 10.0 days (W1) for the five different measurement bursts (consisting of 14 consecutive days). The number of participating adolescents per measurement burst ranged from 97 (W4) to 131 (W1). The average total of completed assessments was 37.6 days (maximum 70 days), based on the entire sample (also see Supplementary Table S1 in the appendices).

#### Boundary diffusion

Daily parental boundary diffusion behaviors were measured separately for mothers and fathers. Based on face validity, 2 triangulation items and 2 emotional parentification items were selected from existing, more extensive instruments (Coparenting Behavior Questionnaire [CBQ]; Schum & Stolberg, 2007; Parentification Questionnaire [PQ]; Hooper & Wallace, 2010). We modified the wording to reflect adolescents’ daily experiences: “Today my mom/dad told me bad things about my dad/mom”, “Today my mom/dad asked me to carry messages or information to my dad/mom”, “Today I felt like I was the only one my mom/dad could turn to (with her/his problems)”, “Today my mom/dad told me about her/his problems (e.g., concerning finances, at work, in their love life, with family members, or with my father/mother)”. All answers were given on a 5-point Likert scale, ranging from (1) *Not (true) at all* to (5) *Very much/Completely true*. Within-dyad and between-dyad correlations between triangulation and parentification were significant both in the daily and half-yearly measures: *r* = 0.26^***^, *r* = 0.68^***^, *r* = 0.46^***^, *r* = 0.64^***^, respectively. Within-dyad factor loadings for the 4 items together ranged from λ = 0.39 to 0.54 for the daily and from λ = 0.49 to 0.74 for the aggregated half-yearly measures.

#### Parent-adolescent warmth & conflict

Daily warmth in the parent-adolescent dyad was measured with adolescents’ perception of parental warmth, using a single item: “Today my mom/dad showed me that she/he cares about me (loves me).” Parent-adolescent conflict was also measured by a single item: “Today my mom/dad and I argued/quarreled.” They answered on a 5-point Likert scale, ranging from (1) *Not at all* to (5) *Very much*. The aggregated daily warmth and conflict measures of measurement bursts 3 and 5 correlated with the more extensive surveys to measure parental warmth and parent-adolescent conflict in the macro questionnaires (i.e., warmth subscale of the CBQ, Schum & Stolberg, 2007; short version of the conflict subscale of the Network Relationship Inventory [NRI], De Goede et al. 2009; Furman & Buhrmester, 1985), that were administered right before adolescents started the daily measures. Correlations ranged from *r* = 0.35^***^ to *r* = 0.64^***^, indicating convergent between-dyad validity.

#### Gender, age, and living arrangement

Information on adolescents’ characteristics and living arrangement were pulled from the macro questionnaires that were administered at the first annual assessment. Most (65.4%) lived with both parents an equal amount of the time (3–4 days/nights each), a third (33.1%) lived entirely or mostly with their mother (minimum of 5 days/nights), and few (1.5%) lived mostly with their father.

### Analyses

To examine the research questions multilevel process analysis was used (Papp, 2004), which is specifically suitable for a focus on within-dyad effects. Because of the relatively small sample size, parent-adolescent warmth and conflict were examined in separate multilevel models, and the associations of boundary diffusion with relationship quality of the adolescent with the actor parent and the other parent were tested separately (4 dependent variables; Hypotheses 1a,b). Both concurrent associations and lagged effects were assessed, on a daily and a half-yearly timescale, resulting in 16 different models. For the models on the daily associations, the daily reports (70 timepoints) were used, and for the models on the half-yearly associations (5 timepoints) the aggregated daily reports per measurement burst were used. In all models, three levels were specified: Within-dyad (level-1), because of repeated measurement occasions within dyads; between-dyad (level-2), consisting of all the different dyads; and between-family (level-3), because of multiple dyads (mother and father) and children nested within families.

In step 1 an unconditional means model was run to identify the amount of variance in warmth and conflict on the different levels [i.e., the intra-class correlation (ICC) or variance partition coefficient (VPC)]. In step 2 warmth/conflict was associated with boundary diffusion at the within-dyad level (level-1) and the between-dyad level (level-2), while accounting for family dependency (level-3). Simultaneously, the study controlled for adolescents’ residence that day or wave (level-1), while also including adolescents’ age, gender, and their living arrangement as level-2 covariates, and time since the divorce as a level-3 covariate. This represents the fixed-effect regression model for the concurrent effects. For both the daily and half-yearly lagged effects on the within-dyad level (level-1), daily warmth/conflict were regressed on boundary diffusion on the previous day or wave (t-1), while controlling for warmth/conflict the previous day or wave. In the models with the day-to-day lagged effects, the large time lag between the different measurement bursts (6 months) was accounted for by omitting the t-1 variable for the first day of each new burst. A visualization of the fixed effect models can be found in the supplementary material (Supplementary Fig. S1).

Step 3 involved adding a random slope for the level-1 regression effects of boundary diffusion on warmth and conflict, to allow for differences between dyads in these effects (random slope model; Hypothesis 2a), and to examine cross-level interactions of level-2 variables (age, gender, and living arrangement, Hypotheses 2b-d) with level-1 boundary diffusion (step 4; Heisig & Schaeffer, 2019). The covariance between the intercept and random slope was included, as recommended by Hox and colleagues (2018). These cross-level interactions were entered for each moderator of within-dyad effects separately. In all models, boundary diffusion on level-1 was centered at the dyad mean, and on level-2 at the family mean, to separate the fluctuations around a dyad’s own mean from differences between dyads (Brincks et al., 2017). Throughout the analyses Bayesian estimation was used (requirement for three-level random models) together with Full Information Maximum Likelihood (FIML) to handle missing data on the item level in *Mplus 8.8* (Muthén & Muthén, 2018). The deviance information criterium (DIC) for each step of all models are found in the appendices, Supplementary Table S2.

## Results

### Descriptive Statistics

The correlations and means of the study variables are depicted in Table 1. Boundary diffusion negatively correlated with warmth from both parents, and positively with parent-adolescent conflict. Warmth and conflict with the actor parent positively correlated with warmth and conflict with the other parent. Warmth and conflict showed a negative correlation.

**Table 1:** Correlations among and descriptive statistics of the study variables

	1.	2.	3.	4.	5.	*M*	*SD*
1. Boundary diffusion actor	–	−0.22^***^	0.55^***^	−0.16^***^	0.33^***^	1.11	0.26
2. Warmth actor	−0.12^***^	–	−0.27^***^	0.70^***^	−0.14^***^	4.17	0.84
3. Conflict actor	0.38^***^	−0.18^***^	–	−0.14^***^	0.34^***^	1.18	0.42
4. Warmth other	−0.12^***^	0.55^***^	−0.10^***^	–	−0.27^***^	4.17	0.84
5. Conflict other	0.27^***^	−0.10^***^	0.21^***^	−0.18^***^	–	1.18	0.42
*M*	1.10	4.21	1.16	4.21	1.16		
*SD*	0.30	1.02	0.53	1.02	0.53		

Below the diagonal are the daily measures, above the diagonal the (aggregated) half-yearly measures. The means and standard deviations are the same for warmth and conflict of either the actor or other parent, because it entails the same variable. Actor or other parent is only in reference to who is showing the boundary diffusion behavior

***p < 0.001

As a first step, the ICC’s of daily warmth and conflict (Table 2) show that over half of the variance was situated at the within-dyad level (level-1), and approximately 20% was at the between-dyad level (level-2). Considerably more variance was located at the between-family level (level-3) for daily parental warmth when compared to parent-adolescent conflict. Most of the variance in warmth and conflict at the half-yearly intervals was again at the within-dyad level (level-1), although relatively less so than in the daily measures. This was to be expected, given that the daily scores were aggregated and thus resulted in fewer timepoints. About a quarter of the variance was at the between-dyad level (level-2), and more variance in warmth than in conflict was found at the between-family level (level-3).

**Table 2:** Variances of the outcome variables on the different levels (step 1)

Step 1	ICC within-dyad (level-1)	ICC between-dyad (level-2)	ICC family (level-3)
Daily warmth	0.53	0.21	0.25
Daily conflict	0.70	0.21	0.09
Half-yearly warmth	0.39	0.26	0.35
Half-yearly conflict	0.52	0.28	0.20

### Between-dyad associations

As shown in Tables 3–6 (step 2), significant between-dyad associations were mainly found for boundary diffusion and parent-adolescent conflict, but not between boundary diffusion and warmth (Hypothesis 1a). In dyads where adolescents generally experienced more boundary diffusion than other dyads, they also reported more conflict with both their parents.

**Table 3:** Concurrent and lagged within-dyad effects for warmth with the actor and other parent on a daily timescale (steps 2–4)

	Actor parent						Other parent					
	Warmth concurrent			Warmth lagged			Warmth concurrent			Warmth lagged		
Step	*b*	*b SE*	*b 95% CI*	*b*	*b SE*	*95% CI*	*b*	*b SE*	*95% CI*	*b*	*b SE*	*95% CI*
2. Fixed model												
Lev-1: Boundary diffusion	**−0.11**	0.04	**[**−**0.19** −**0.04]**	−**0.10**	0.04	**[**−**0.18** −**0.03]**	−0.02	0.04	[−0.10 0.07]	0.00	0.05	[−0.10 0.10]
Lev-1: Residence other	**−0.26**	0.03	**[**−**0.31** −**0.21]**	−**0.21**	0.03	**[**−**0.26** −0.**16]**	**0.25**	0.03	**[0.19 0.31]**	**0.20**	0.03	**[0.14 0.26]**
Lev-1: Residence switch	**−**0.03	0.02	[−0.08 0.01]	**−0.06**	0.03	**[**−**0.10** −**0.01]**	**0.23**	0.03	**[0.18 0.29]**	**0.12**	0.03	**[0.06 0.19]**
Lev-1: Warmth t-1				**0.28**	0.01	**[0.26 0.31]**				**0.26**	0.02	**[0.23 0.29]**
Lev-2: Boundary diffusion	−0.16	0.19	[−0.54 0.21]	−0.24	0.17	[−0.56 0.09]	−0.13	0.19	[−0.51 0.24]	**−0.24**	0.16	**[−0.74 −0.01]**
Lev-2: Age	−**0.07**	0.03	**[**−**0.12** −**0.02]**	**−0.07**	0.03	**[−0.12 −0.02]**	−**0.06**	0.03	**[**−**0.12** −**0.01]**	−**0.07**	0.03	**[**−**0.12** −**0.01]**
Lev-2: Gender	0.13	0.08	[−0.04 0.28]	0.11	0.09	[−0.06 0.28]	0.13	0.09	[−0.04 0.30]	0.08	0.09	[−0.08 0.27]
Lev-2: Living other	−0.14	0.11	[−0.35 0.07]	−0.08	0.12	[−0.31 0.15]	0.16	0.12	[−0.07 0.38]	0.11	0.12	[−0.15 0.33]
Lev-2: Living shared	0.15	0.13	[−0.10 0.39]	0.13	0.13	[−0.13 0.38]	0.27	0.13	[−0.01 0.54]	0.24	0.14	[−0.05 0.51]
Lev-3: Time divorce	0.01	0.01	[−0.02 0.03]	0.01	0.01	[−0.02 0.03]	0.01	0.01	[−0.02 0.03]	0.01	0.01	[−0.02 0.03]
3. Random slope model												
Lev-2: Warm <> slope BD	0.01	0.03	[−0.06 0.08]	**0.06**	0.01	**[0.01 0.12]**	0.04	0.03	[−0.02 0.10]	−0.01	0.04	[−0.09 0.06]
Lev-2: Variance slope BD	**0.26**	0.08	**[0.14 0.45]**	**0.09**	0.05	**[0.02 0.21]**	**0.07**	0.05	**[0.02 0.19]**	**0.19**	0.08	**[0.07 0.39]**
Lev-3: Variance slope BD	**0.05**	0.05	**[0.00 0.19]**	**0.05**	0.04	**[0.01 0.15]**	**0.07**	0.06	**[0.01 0.21]**	**0.04**	0.04	**[0.00 0.16]**
4. Interactions												
a. Cross: Age*slope BD	−0.02	0.06	[−0.14 0.10]	0.01	0.05	[−0.09 0.12]	−0.03	0.06	[−0.14 0.09]	−0.06	0.07	[−0.20 0.08]
b. Cross: Gend*slope BD	0.12	0.14	[−0.16 0.41]	0.14	0.13	[−0.11 0.38]	−0.07	0.13	[−0.34 0.18]	−0.22	0.16	[−0.54 0.09]
c. Cross: Other*slope BD	0.25	0.24	[−0.23 0.72]	0.01	0.22	[−0.40 0.46]	0.17	0.21	[−0.24 0.58]	−0.23	0.28	[−0.77 0.34]
c. Cross: Shared*slope BD	0.14	0.19	[−0.24 0.52]	−0.08	0.16	[−0.39 0.23]	0.10	0.19	[−0.29 0.49]	−0.30	0.25	[−0.77 0.21]

Estimates printed in bold are significant: 95% CI does not include 0. In step 2, the within-dyad effect of boundary diffusion on warmth (lev-1) is the pooled effect of all dyads. In steps 3 and 4, this effect (slope) was allowed to vary across dyads and families. For residence (lev-1), reference group = house actor parent, residence other = at home with other parent, residence switch = adolescent switched houses that day; For gender (lev-2), reference group = boys; For living arrangement (lev-2), reference group = mostly living with actor parent

*Lev* Level, *Slope BD* Random slope of within-dyad boundary diffusion on warmth, *Cross* Cross-level interaction, *Gend* Gender

**Table 4:** Concurrent and lagged within-dyad effects for conflict with the actor and other parent on a daily timescale (steps 2–4)

	Actor parent						Other parent					
	Concurrent conflict			Lagged conflict			Concurrent conflict			Lagged conflict		
Step	*b*	*b SE*	*95% CI*	*b*	*b SE*	*95% CI*	*b*	*b SE*	*95% CI*	*b*	*b SE*	*95% CI*
2. Fixed model												
Lev-1: Boundary diffusion	**0.56**	0.02	**[0.52 0.60]**	**0.15**	0.03	**[0.10 0.20]**	**0.22**	0.02	**[0.17 0.26]**	0.04	0.03	[−0.02 0.10]
Lev-1: Residence other	−**0.11**	0.01	**[**−**0.14** −**0.09]**	−**0.11**	0.02	**[**−**0.14** −**0.08]**	**0.14**	0.02	**[0.19 0.31]**	**0.12**	0.02	**[0.08 0.15]**
Lev-1: Residence switch	−**0.05**	0.01	**[**−**0.07** −**0.02]**	−**0.05**	0.02	**[**−**0.08** −**0.02]**	**0.09**	0.02	**[0.18 0.29]**	**0.07**	0.02	**[0.03 0.11]**
Lev-1: Conflict t-1				**0.16**	0.01	**[0.13 0.18]**				**0.16**	0.02	**[0.13 0.19]**
Lev-2: Boundary diffusion	**1.0**	0.09	**[0.83 1.2]**	**1.0**	0.08	**[0.86 1.2]**	**0.85**	0.09	**[0.66 1.0]**	**0.63**	0.08	**[0.46 0.79]**
Lev-2: Age	−0.02	0.01	[−0.04 0.01]	−0.02	0.01	[−0.04 0.01]	−0.01	0.01	[−0.04 0.01]	−0.01	0.02	[−0.04 0.02]
Lev-2: Gender	0.00	0.03	[−0.07 0.07]	−0.01	0.04	[−0.08 0.07]	0.02	0.04	[−0.06 0.09]	0.02	0.04	[−0.07 0.11]
Lev-2: Living other	0.06	0.05	[−0.04 0.15]	0.05	0.06	[−0.06 0.16]	−0.08	0.06	[−0.19 0.03]	−0.08	0.07	[−0.21 0.04]
Lev-2: Living shared	0.04	0.05	[−0.06 0.14]	0.06	0.05	[−0.05 0.16]	−0.04	0.06	[−0.15 0.07]	−0.03	0.07	[−0.15 0.11]
Lev-3: Time divorce	0.00	0.00	[−0.01 0.01]	0.00	0.01	[−0.01 0.01]	0.00	0.00	[−0.01 0.01]	0.00	0.01	[−0.01 0.01]
3. Random slope model												
Lev-2: Conf. <> slope BD	**0.07**	0.01	**[0.05 0.09]**	**0.02**	0.01	**[0.00 0.04]**	0.00	0.01	[−0.02 0.02]	−0.01	0.01	[−0.03 0.02]
Lev-2: Variance slope BD	**0.23**	0.05	**[0.15 0.34]**	**0.11**	0.03	**[0.06 0.19]**	**0.06**	0.03	**[0.02 0.13]**	**0.11**	0.05	**[0.04 0.23]**
Lev-3: Variance slope BD	**0.07**	0.04	**[0.01 0.18]**	**0.04**	0.03	**[0.00 0.11]**	**0.13**	0.05	**[0.06 0.25]**	**0.07**	0.05	**[0.01 0.19]**
4. Interactions												
a. Cross: Age*slope BD	0.06	0.04	[−0.03 0.14]	−0.02	0.04	[−0.10 0.06]	0.07	0.04	[−0.01 0.15]	0.05	0.05	[−0.04 0.15]
b. Cross: Gend*slope BD	−0.04	0.11	[−0.26 0.18]	−0.14	0.10	[−0.34 0.06]	−0.09	0.11	[−0.29 0.12]	−0.08	0.12	[−0.30 0.17]
c. Cross: Other*slope BD	−0.08	0.18	[−0.43 0.27]	0.02	0.17	[−0.31 0.36]	0.09	0.15	[−0.19 0.38]	0.17	0.18	[−0.20 0.54]
c. Cross: Shared*slope BD	−0.07	0.15	[−0.37 0.23]	0.11	0.13	[−0.13 0.37]	0.17	0.15	[−0.13 0.47]	0.34	0.18	[−0.03 0.70]

Estimates printed in bold are significant: 95% CI does not include 0. In step 2, the within-dyad effect of boundary diffusion on conflict (lev-1) is the pooled effect of all dyads. In steps 3 and 4, this effect (slope) was allowed to vary across dyads and families. For residence (lev-1), reference group = house actor parent, residence other = at home with other parent, residence switch = adolescent switched houses that day; For gender (lev-2), reference group = boys; For living arrangement (lev-2), reference group = mostly living with actor parent

*Lev* Level, *Slope BD* Random slope of within-dyad boundary diffusion on conflict, *Cross* Cross-level interaction, *Gend* Gender

**Table 5:** Concurrent and lagged within-dyad effects for warmth with the actor and other parent on a half-yearly timescale (steps 2–4)

	Actor parent						Other parent					
	Warmth concurrent			Warmth lagged			Warmth concurrent			Warmth lagged		
Step	*b*	*b SE*	*95% CI*	*b*	*b SE*	*95% CI*	*b*	*b SE*	*95% CI*	*b*	*b SE*	*95% CI*
2. Fixed model												
Lev-1: Boundary diffusion	−**0.45**	0.10	**[**−**0.64** −**0.26]**	−0.07	0.10	[−0.28 0.13]	−0.11	0.10	[−0.30 0.09]	−0.18	0.10	[−0.38 0.02]
Lev-1: Residence actor	**0.31**	0.09	**[0.13 0.48]**	**0.39**	0.10	**[0.19 0.58]**	−**0.36**	0.10	**[**−**0.55** −**0.17]**	−**0.39**	0.10	**[**−**0.59** −**0.18]**
Lev-1: Warmth t-1				−0.04	0.04	[−0.12 0.05]				−0.05	0.04	[−0.13 0.03]
Lev-2: Boundary diffusion	−0.32	0.20	[−0.71 0.07]	**−0.59**	0.20	**[**−**0.99** −**0.19]**	−0.26	0.19	[−0.65 0.14]	**−0.46**	0.20	**[−0.87 −0.06]**
Lev-2: Age	**−0.06**	0.03	**[**−**0.12** −**0.01]**	−**0.08**	0.03	**[**−**0.14** −**0.02]**	**−0.07**	0.03	**[**−**0.12** −**0.02]**	**−0.08**	0.03	**[−0.13 −0.02]**
Lev-2: Gender	0.09	0.08	[−0.07 0.25]	0.13	0.09	[−0.04 0.30]	0.09	0.09	[−0.07 0.26]	0.12	0.09	[−0.05 0.30]
Lev-2: Living other	**−**0.09	0.12	[−0.33 0.14]	**−**0.02	0.14	[−0.29 0.25]	0.07	0.12	[−0.18 0.31]	0.03	0.14	[−0.24 0.30]
Lev-2: Living shared	0.18	0.13	[−0.07 0.44]	0.15	0.14	[−0.12 0.43]	0.25	0.13	[−0.01 0.51]	0.18	0.14	[−0.11 0.45]
Lev-3: Time divorce	0.00	0.01	[−0.02 0.03]	−0.01	0.01	[−0.03 0.01]	0.00	0.01	[−0.01 0.03]	−0.01	0.01	[−0.03 0.01]
3. Random slope model												
Lev-2: Warm <> slope BD	0.15	0.08	[−0.01 0.32]	0.04	0.08	[−0.12 0.20]	**0.19**	0.10	**[0.01 0.39]**	0.03	0.09	[−0.14 0.22]
Lev-2: Variance slope BD	**0.30**	0.23	**[0.04 0.93]**	**0.15**	0.15	**[0.02 0.58]**	**0.41**	0.30	**[0.06 1.2]**	**0.14**	0.19	**[0.02 0.71]**
Lev-3: Variance slope BD	**0.61**	0.40	**[0.12 1.7]**	**0.14**	0.18	**[0.01 0.68]**	**0.51**	0.44	**[0.06 1.7]**	**0.21**	0.30	**[0.01 1.0]**
4. Interactions												
a. Cross: Age*slope BD	0.10	0.17	[−0.23 0.42]	0.20	0.15	[−0.09 0.48]	−0.04	0.17	[−0.38 0.30]	0.18	0.17	[−0.17 0.51]
b. Cross: Gend*slope BD	0.21	0.39	[−0.60 0.95]	0.35	0.33	[−0.26 1.0]	0.22	0.42	[−0.64 1.0]	0.48	0.36	[−0.21 1.2]
c. Cross: Other*slope BD	0.30	0.55	[−0.78 1.4]	0.21	0.55	[−0.89 1.3]	−0.02	0.61	[−1.2 1.2]	−0.73	0.60	[−1.9 0.46]
c. Cross: Shared*slope BD	0.36	0.54	[−0.65 1.4]	−0.37	0.48	[−1.3 0.62]	−0.27	0.59	[−1.4 0.91]	−0.91	0.58	[−2.1 0.21]

Estimates printed in bold are significant: 95% CI does not include 0. In step 2, the within-dyad effect of boundary diffusion on warmth (lev-1) is the pooled effect of all dyads. In steps 3 and 4, this effect (slope) was allowed to vary across dyads and families. For gender (lev-2), reference group = boys; For living arrangement (lev-2), reference group = mostly living with actor parent

*Lev* Level, *Slope BD* Random slope of within-dyad boundary diffusion on warmth, *Cross* Cross-level interaction, *Gend* Gender

**Table 6:** Concurrent and lagged within-dyad effects for conflict with the actor and other parent on a half-yearly timescale (steps 2–4)

	Actor parent						Other parent					
	Concurrent conflict			Lagged conflict			Concurrent conflict			Lagged conflict		
Step	*b*	*b SE*	*95% CI*	*b*	*b SE*	*95% CI*	*b*	*b SE*	*95% CI*	*b*	*b SE*	*95% CI*
2. Fixed model												
Lev-1: Boundary diffusion	**0.84**	0.05	**[0.74 0.94]**	−0.09	0.08	[−0.24 0.07]	**0.29**	0.06	**[0.18 0.41]**	0.13	0.07	[−0.01 0.26]
Lev-1: Residence actor	0.09	0.05	[−0.00 0.18]	0.06	0.06	[−0.05 0.19]	−0.02	0.06	[−0.13 0.09]	−0.03	0.06	[−0.16 0.10]
Lev-1: Conflict t-1				−0.08	0.05	[−0.18 0.02]				−**0.12**	0.04	**[**−**0.21 0.03]**
Lev-2: Boundary diffusion	**0.96**	0.09	**[0.78 1.2]**	**1.0**	0.10	**[0.80 1.2]**	**0.49**	0.11	**[0.27 0.71]**	**0.59**	0.12	**[0.36 0.81]**
Lev-2: Age	−0.02	0.01	[−0.04 0.01]	−0.01	0.01	[−0.03 0.02]	−0.01	0.02	[−0.04 0.02]	−0.01	0.02	[−0.04 0.02]
Lev-2: Gender	0.00	0.04	[−0.08 0.08]	−0.04	0.04	[−0.12 0.04]	−0.01	0.04	[−0.09 0.08]	−0.03	0.05	[−0.12 0.06]
Lev-2: Living other	0.06	0.07	[−0.05 0.18]	0.05	0.06	[−0.07 0.18]	−0.07	0.07	[−0.21 0.07]	−0.08	0.08	[−0.24 0.07]
Lev-2: Living shared	0.03	0.06	[−0.09 0.14]	0.04	0.07	[−0.09 0.18]	−0.04	0.06	[−0.16 0.09]	−0.00	0.07	[−0.15 0.14]
Lev-3: Time divorce	0.00	0.01	[−0.01 0.01]	0.00	0.01	[−0.01 0.01]	0.00	0.00	[−0.01 0.01]	0.00	0.01	[−0.01 0.01]
3. Random slope model												
Lev-2: Conf. <> slope BD	**0.06**	0.02	**[0.02 0.10]**	−0.00	0.02	[−0.04 0.04]	0.04	0.02	[−0.01 0.09]	0.01	0.03	[−0.05 0.06]
Lev-2: Variance slope BD	**0.21**	0.13	**[0.06 0.54]**	**0.05**	0.06	**[0.01 0.23]**	**0.13**	0.11	**[0.02 0.45]**	**0.09**	0.15	**[0.01 0.55]**
Lev-3: Variance slope BD	**0.65**	0.28	**[0.26 1.4]**	**0.09**	0.10	**[0.01 0.37]**	**0.16**	0.12	**[0.02 0.46]**	**0.23**	0.27	**[0.02 1.0]**
4. Interactions												
a. Cross: Age*slope BD	0.02	0.10	[−0.18 0.21]	−0.12	0.10	[−0.32 0.08]	0.01	0.10	[−0.18 0.21]	−0.12	0.12	[−0.36 0.12]
b. Cross: Gend*slope BD	0.36	0.27	[−0.16 0.90]	−0.14	0.22	[−0.56 0.29]	−0.19	0.23	[−0.62 0.27]	0.02	0.30	[−0.58 0.58]
c. Cross: Other*slope BD	0.12	0.33	[−0.54 0.76]	−0.16	0.39	[−0.90 0.64]	0.04	0.35	[−0.60 0.76]	0.21	0.49	[−0.72 1.2]
c. Cross: Shared*slope BD	−0.01	0.38	[−0.72 0.79]	−0.09	0.32	[−0.71 0.55]	0.41	0.31	[−0.18 1.0]	0.49	0.47	[−0.46 1.4]

Estimates printed in bold are significant: 95% CI does not include 0. In step 2, the within-dyad effect of boundary diffusion on conflict (lev-1) is the pooled effect of all dyads. In steps 3 and 4, this effect (slope) was allowed to vary across dyads and families. For gender (lev-2), reference group = boys; For living arrangement (lev-2), reference group = mostly living with actor parent

*Lev* Level, *Slope BD* Random slope of within-dyad boundary diffusion on conflict, *Cross* Cross-level interaction, *Gend* Gender

### Within-dyad Effects

#### Daily interval

First, significant concurrent and longitudinal within-dyad effects were found for daily warmth and conflict with the actor parent (Tables 3–4; step 2; Hypothesis 2b). This means that when a parent showed more daily boundary diffusion than usual, adolescents experienced a decrease in warmth and an increase in conflict with that specific parent, both the same and the next day. Second, on days when adolescents experienced increased boundary diffusion by one of their parents (actor), they also had more conflict with the other parent that same day. Boundary diffusion by the actor parent did not predict changes in their level of conflict with the other parent the next day, nor warmth from the other parent on either days. The concurrent associations seemed stronger than the lagged effects, but this could not be tested explicitly since separate models were used for estimating the concurrent and lagged effects. Third, adolescents generally reported more daily warmth and conflict with a parent when they were at that parent’s house, and younger adolescents reported more warmth than older adolescents. Gender, living arrangement, and time since divorce did not significantly relate to daily warmth or conflict.

#### Half-yearly interval

Adolescents who reported more boundary diffusion than usual also experienced more conflict with both parents that same period of two weeks, and less warmth from the actor parent (Tables 5 and 6; step 2). Boundary diffusion did not significantly predict changes in warmth or conflict with either one of the parents six months later (Hypothesis 1c). When visually inspecting the individual slopes of all dyads more closely (Supplemetary Figs. S2–S17 in the Appendices), associations mostly seemed to reflect a negative impact of boundary diffusion on the relationship quality with both parents, but especially with the other parent. The confidence intervals of these half-yearly within-person effects are not symmetrical around 0, which given the Bayesian estimation can be tentatively interpreted as an indication for long-term effects (e.g., Kruschke & Liddel, 2018).

Mostly in line with the daily results, adolescents generally reported more warmth from a specific parent when they spent more time at that parent’s house those two weeks, whereas (aggregated) parent-child conflict was not linked to the time spent at a certain house. Younger adolescents reported more warmth. Gender, living arrangement, and time since divorce did not significantly relate to half-yearly warmth or conflict.

### Heterogeneity

In addition to the fixed within-dyad effects (i.e., representing the pooled effects across all dyads), heterogeneity was found between dyads and families in their associations of daily boundary diffusion with both warmth and conflict (step 3; Hypothesis 2a). The random slope variances were significant in all models at the between-dyad (level-2) and family (level-3) level, yet some of these variances were close to zero, suggesting little heterogeneity. Despite considerable heterogeneity, the random slopes were not significantly related to age, gender, or living arrangement (Tables 3–6; step 4). This means that none of the examined moderators explained the heterogeneity in within-dyad effects (Hypotheses 2b-d).

## Discussion

Boundary diffusion is a particular risk for adolescents with divorced parents (Perrin et al., 2013; Rowen & Emery, 2018), and likely to affect their relationship quality. Prior research does not provide clear support for either the alienation or conflict perspective. The current study extended previous work by examining concurrent and longitudinal links between post-divorce boundary diffusion and parent-adolescent relationship quality (warmth and conflict), both on the short- and the long-term, and between-dyad associations were separated from within-dyad effects. The findings mostly support the conflict perspective, both between and within parent-adolescent dyads. Between dyads, those adolescents who experienced more boundary diffusion than others also reported more conflict with both their parents. Within dyads, when adolescents experienced more daily boundary diffusion than usual by one of their parents, there was a decrease in daily relationship quality with this specific parent both the same and the next day. Boundary diffusion affected the relationship with the other parent to a much lesser degree, and there were no significant long-term associations.

### Boundary Diffusion and Parent-adolescent Relationship Quality: between-dyad Associations

When adolescents reported more boundary diffusion than others, their post-divorce relationship quality with both parents seems under pressure, as they engaged in more conflict. Less support was found for between-dyad associations of boundary diffusion with parent-child warmth (i.e., in 3 of 8 models). This suggests that post-divorce boundary diffusion is a risk factor for parent-adolescent conflict particularly, which aligns with the conflict perspective and with previous studies that reported a negative association between boundary diffusion and parent-child relationship quality (Fosco & Grych, 2010; Rowen & Emery, 2018). Whereas previous findings mainly addressed parent-adolescent warmth or closeness (Rowen & Emery 2014, 2019), the current findings on the between-dyad level predominantly pertain to conflict. Future research is warranted to gain a better understanding of the association between boundary diffusion and these different aspects of the parent-adolescent relationship.

### Boundary Diffusion and Parent-adolescent Relationship Quality: Within-dyad Associations

Most of the within-dyad effects again substantiated the conflict perspective, yet with more evidence found on the daily than on the half-yearly timescale. When adolescents experienced more boundary diffusion than usual from a particular parent, they reported decreased warmth from and increased conflict with that specific parent the same day, as well as the next day. More daily boundary diffusion by the actor parent was only related to more conflict between the adolescent and the other parent that same day but not the next day, nor to experienced warmth from the other parent. These within-dyad findings align with a “boomerang” effect as described by Rowen and Emery (2019): Parents’ attempts to form a parent-child alliance appear to “backfire” for that specific parent. Although there was significant variation in all effects for the different dyads and families, when visually inspecting the dyads’ individual slopes, in only few dyads boundary diffusion was clearly linked to a better relationship quality with that specific parent (i.e., alienation perspective). The blurring of hierarchical boundaries between parents and their adolescents seems to negatively affect the day-to-day relationship quality of most parent-adolescent dyads. Given that boundary diffusion behaviors are likely due to changes and issues in the interparental subsystem following divorce, in line with a family systems approach (e.g., Cox & Paley, 1997), the findings may be considered in light of spillover dynamics within the family. Also, the findings emphasize the relevance of maintaining hierarchical boundaries after divorce.

In contrast to the findings on a daily level, boundary diffusion did not significantly affect adolescents’ relationship quality with either parent six months later. This seems to suggest that these daily dynamics between parents and adolescents are not translated to more long-term relationship quality. Perhaps the daily interactions were not as impactful to also have a lasting effect on the relationship longer term. This could reflect the relatively low levels of boundary diffusion in this sample. Instead of an actual absence of long-term effects, the non-significant findings could potentially reflect a lack of sufficient power on the within-dyad level as there were only five (aggregated) timepoints per dyad. Visual inspection of the individual slopes and the asymmetrical confidence intervals of the estimated (fixed) effects did hint towards deteriorated parent-child relationship quality with the other parent when adolescents experienced more boundary diffusion than usual six months prior. This tentative indication for long-term effects of boundary diffusion should be interpreted with caution and future research is needed to further examine these associations more thoroughly, including individual differences in the potential long-term impact of boundary diffusion.

### Adolescent Age, Gender, and Post-divorce Living Arrangements

None of the moderators – adolescent age, gender, or post-divorce living arrangement – significantly explained the variance in within-dyad associations, which is in contrast with the hypotheses. Although this could be due to potential power issues in finding small moderation effects, the selected moderators in itself may have been too simplistic. In regard to adolescents’ gender, it might be important to additionally consider whether it involves the relationship with either their mother or father (e.g., Van Lissa et al. 2019). Despite the theoretical considerations that guided the expectations, in hindsight, adolescents’ gender and age may have been too generic to explain differences in dyadic fluctuations. Gender differences may be more complex than girls just being more sensitive to social relations, and the differences across the group of girls are likely more profound than differences between boys and girls perse. There may also be large variability in cognitive maturation among adolescents, despite a general increase in social-cognitive skills with age. Exploring other characteristics that more directly measure adolescents’ interpretation of parental behaviors, could perhaps offer a better assessment of their vulnerability to parent-adolescent dynamics and boundary diffusion in particular. Highly empathic children have been found to be more vulnerable to boundary diffusion after divorce than those with low empathy (Van Dijk et al., 2022), potentially reflecting a more nuanced assessment of how adolescents signal, interpret, and internalize parental behaviors. Future research on the role of adolescents’ empathy in explaining differences in within-dyad dynamics, could help in unraveling the complex dynamics relevant for adolescent well-being post-divorce. In regard to their living arrangement, the sample may have lacked diversity, as prior studies that found (partial) support for the alienation perspective were based on samples of mothers that had sole custody of the children (Afifi & McManus, 2010; Kenyon & Koerner, 2008). In the current sample most adolescents spent about the same number of days with parents or they structurally saw their nonresident parent at least one or two days a week.

### Limitations and Strengths

Despite the study’s contribution to existent literature on parent-adolescent relationships after divorce, some issues in regard to the diversity of the sample should be addressed. The sample mainly consisted of divorced parents with a medium to high socio-economic status and White, ethnic majority families. As many family studies suffer from these limitations (Fakkel et al., 2020), conducting similar research with more diverse samples is needed. In addition, it was a relatively small and well-functioning sample, with low levels of daily boundary diffusion and parent-adolescent conflict. Although effects may turn out stronger in more high-conflict samples, other patterns could also emerge when the pressure put on an adolescent becomes more excessive. The current sample size prohibited from testing bidirectional effects, and these should be addressed in future research on boundary diffusion in the context of divorce. If the increased conflict in dyads portrays adolescents’ dissatisfaction with parental boundary diffusion, this returns the question as to whether these arguments are effective. Does increased conflict in the parent-adolescent relationship predict decreased boundary diffusion within those dyads? This also raises the issue of potential spillover processes between siblings in divorced families, in which for example negative dynamics in one parent-child dyad might spill over to other dyads in the family, including other parent-child and sibling relationships. Such spillover processes should be addressed in future studies. Although in the current study most variance in the outcome measures was found at the within-dyad level, considerable variance was situated at the family-level and could reflect spillover amongst the different family subsystems.

Although the use of daily measures in the current study gives a unique perspective on post-divorce family functioning on a daily timescale, and reduces potential recall bias in the long-term measures by aggregating these scores (e.g., Repetti et al., 2015), a downside of daily measures is the limited number of items that are available to capture certain constructs. Boundary diffusion is a rather broad construct (e.g., Kerig, 2005), and might have been assessed more accurately with a larger number of items. The limited number of items also prohibited us from making a distinction between parentification and triangulation behaviors by parents, which may yield different findings if examined separately. Lastly, daily data were only available from adolescents themselves. Although this provides valuable information on their specific experiences, it also increases the likeliness of mono-reporter bias. Future research that obtains daily data from both adolescents and their parents is warranted.

Notwithstanding these limitations, the intensive longitudinal design with recently divorced families contributed to a better understanding of boundary diffusion in divorced family’s daily dynamics, as well as on the long-term. Adding to previous work, a particular strength was the current focus on within-dyad effects. As these effects pertain to changes in a specific parent-adolescent dyad, this approach better fits the ideas on the impact of boundary diffusion as postulated in the alienation versus conflict perspective, and in more general theories on family dynamics (Branje et al., 2014; Keijsers & Van Roekel, 2018).

## Conclusion

Cross-generational boundary diffusion is a particular risk for adolescents with separated parents and likely affects their relationship quality. Because these dynamics are expected to occur within specific parent-adolescent dyads, the current study extended previous research by discerning within-dyad effects from between-dyad associations. It further assessed both daily and half-yearly associations, as parent-adolescent dynamics unfold on a daily basis and are considered to drive long-term development. Between dyads, adolescents who experienced more boundary diffusion than others tended to argue more with both their parents after divorce. This warrants practitioners’ attention in identifying parents and adolescents who are at particular risk for conflict after divorce. Within dyads, when a parent showed more daily boundary diffusion than usual, there was a decrease in daily relationship quality with this specific parent, but hardly in the relationship with the other parent. So from day-to-day, boundary diffusion was linked to a deteriorated parent-adolescent relationship quality, especially with the parent that triangulated or parentified them. These daily dynamics between parents and their adolescents are particularly relevant in light of intervention efforts aimed at improving relationship quality after divorce, through altering the day-to-day interactions between parents and adolescents. Long-term associations between boundary diffusion and parent-adolescent relationship quality however need further study, and it should be acknowledged that all associations varied among different parent-adolescent dyads. Neither adolescents’ age, gender, or post-living situation could explain this heterogeneity in effects.

## Supplementary information

Supplementary Information

## Footnotes

1 It should be noted that the term alienation is only used here to describe a theoretical perspective that may explain potential dynamics in parent-adolescent warmth and conflict. This study refrains from the controversial construct of parental alienation (syndrome), where the behavior of an alienating parent is inextricably connected to the child’s rejection of the other parent (e.g., Rowen & Emery, 2018), and is often described in extreme cases of conflict and complex divorces.
