# Supplementary material for: Daily and Half-yearly Associations between Boundary Diffusion and Parent-adolescent Relationship Quality after Divorce
Source: J Youth Adolesc. 2024 Sep 9;54(2):383–99. doi: 10.1007/s10964-024-02064-w (PMC11807060; doi:10.1007/s10964-024-02064-w)

Supplementary Material of **‘Daily and Half-yearly Associations between Boundary Diffusion and Parent-adolescent Relationship Quality after Divorce’** in the Journal of Youth and Adolescence

Table S1. *Descriptive Information on Datapoints per Measurement Burst*

|  | *N* | Datapoints | *M* days per child | *%* |
| --- | --- | --- | --- | --- |
| Wave 1 (0 months)  Wave 2 (6 months)  Wave 3 (12 months)  Wave 4 (18 months)  Wave 5 (24 months) | 131  113  116  97  100 | 1,311  1,035  1,008  831  817 | 10.0  9.2  8.7  8.6  8.2 | 71.5  65.4  62.1  61.2  58.4 |

Table S2. *Deviance Information Criteria (DIC) for the Different Models and Outcomes*

|  | Actor parent | | | |  | Other parent | | | |
| --- | --- | --- | --- | --- | --- | --- | --- | --- | --- |
|  | Warm  concur | Warm lagged | Conf  concur | Conf lagged |  | Warm  concur | Warm lagged | Conf  concur | Conf lagged |
| Daily models  1. Empty model  2. Fixed effects  3. Random slopes  4a. Int: Age  4b. Int: Sex  4c. Int: Living  Half-yearly  1. Empty model  2. Fixed effects  3. Random slopes  4a. Int: Age  4b. Int: Sex  4c. Int: Living | 18,445  18,117  17,951  17,952  17,952  17,954  1,932  1,834  1,851  1,854  1,854  1,854 | 13,986  13,894  13,895  13,891  13,896  1,229  1,230  1,230  1,231  1,232 | 10,604  11,195  9,162  9,162  9,163  9,163  774  998  196  198  196  197 | 7,668  7,225  7,226  7,223  7,226  495  490  491  492  493 |  | 13,382  13,353  13,355  13,355  13,355  1,901  1,869  1,871  1,871  1,871 | 9,820  9,788  9,788  9,787  9,788  1,207  1,205  1,205  1,208  1,209 | 7,167  6,827  6,825  6,827  6,826  743  598  599  598  599 | 5,201  5,094  5,092  5,095  5,094  466  466  467  467  469 |
| *Note.* For all models a minimum of 50,000 and a maximum of 1,000,000 iterations was used, with a PSR convergence criterion of < .0005 (Gelman & Rubin, 1992). Abbreviations: Int = Cross-level interaction term model; Living = Living arrangement. | | | | | | | | | |

*
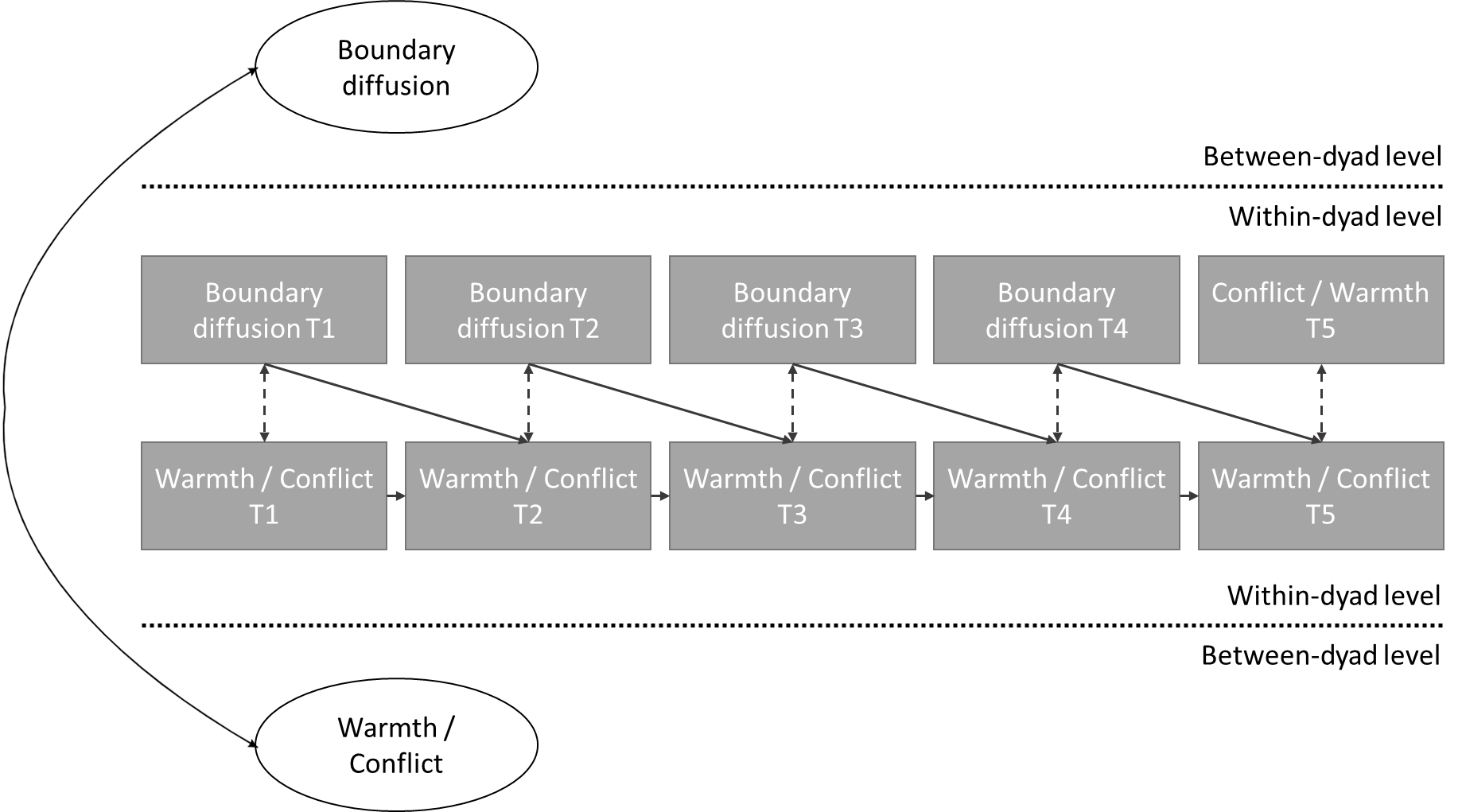
Figure S1.* Visualization of the analytic approach for the long-term models. The daily models are similar yet containing more timepoints. The dashed arrows represent the associations in the concurrent models. The solid arrows represent the associations in the lagged models, including the autoregressive effect for warmth/conflict.

*Figures S2-S9.* Dyad’s daily slopes (i.e., combining unique family slopes and unique dyad slopes). Red refers to worse and green to better relationship quality.


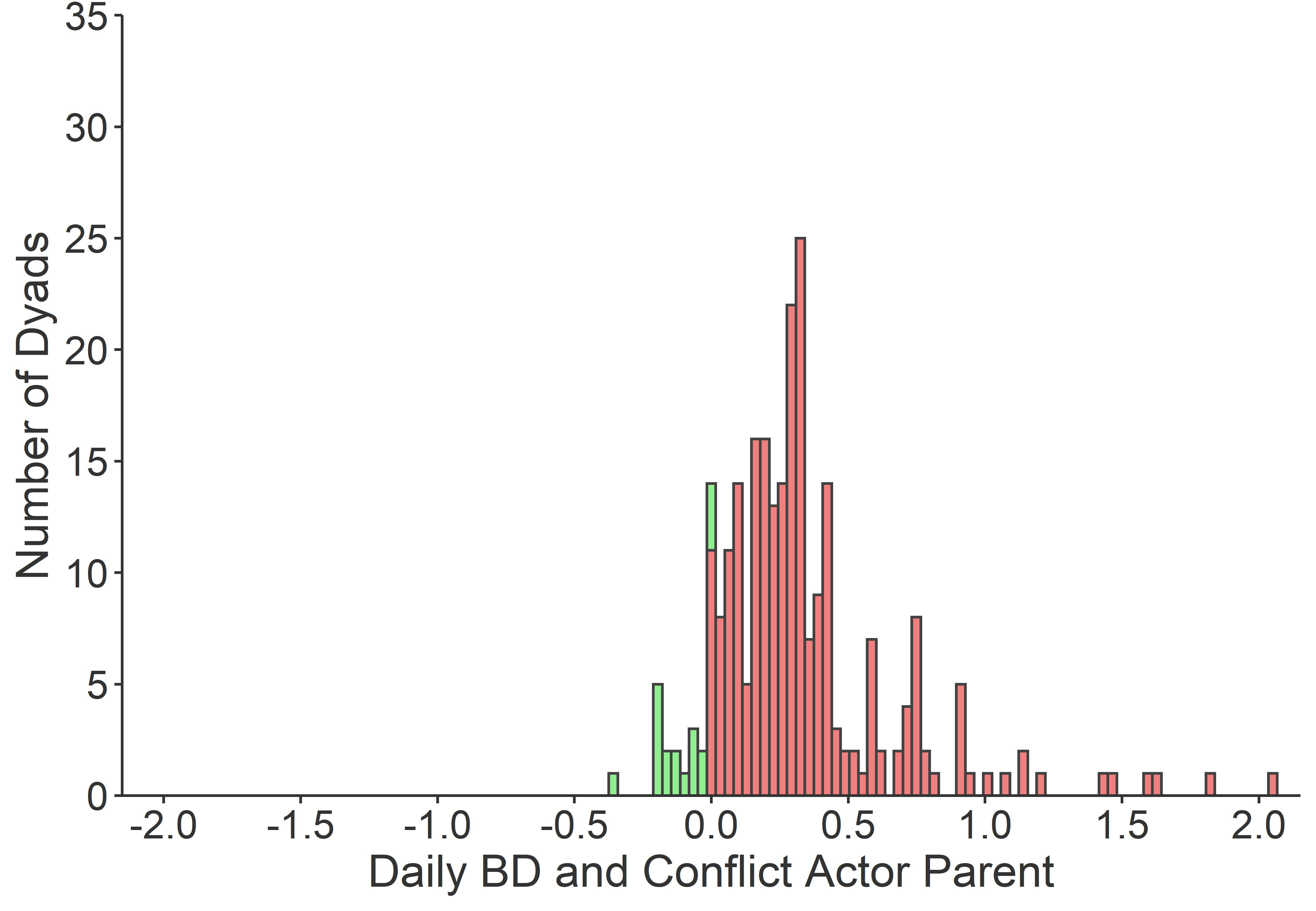

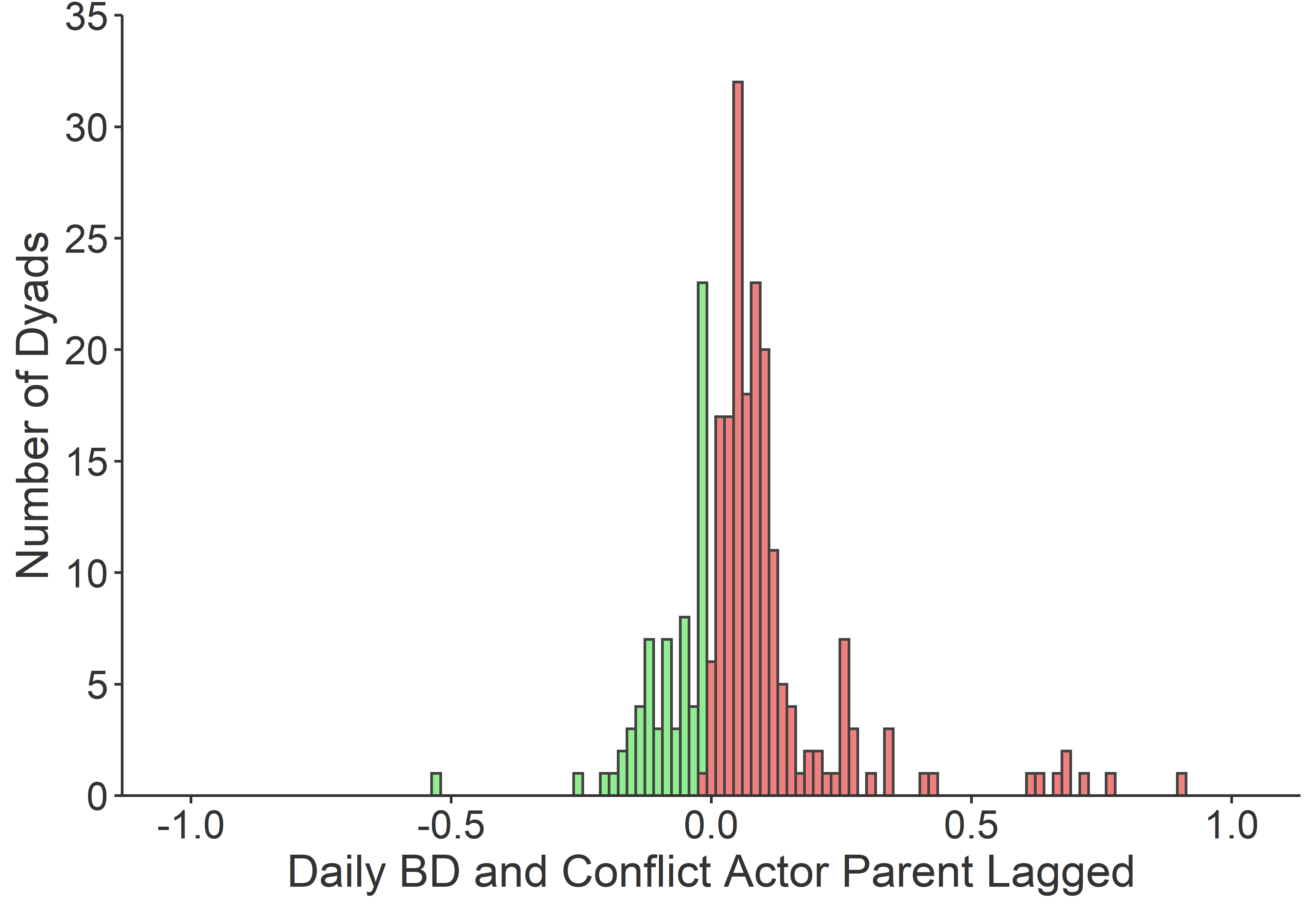

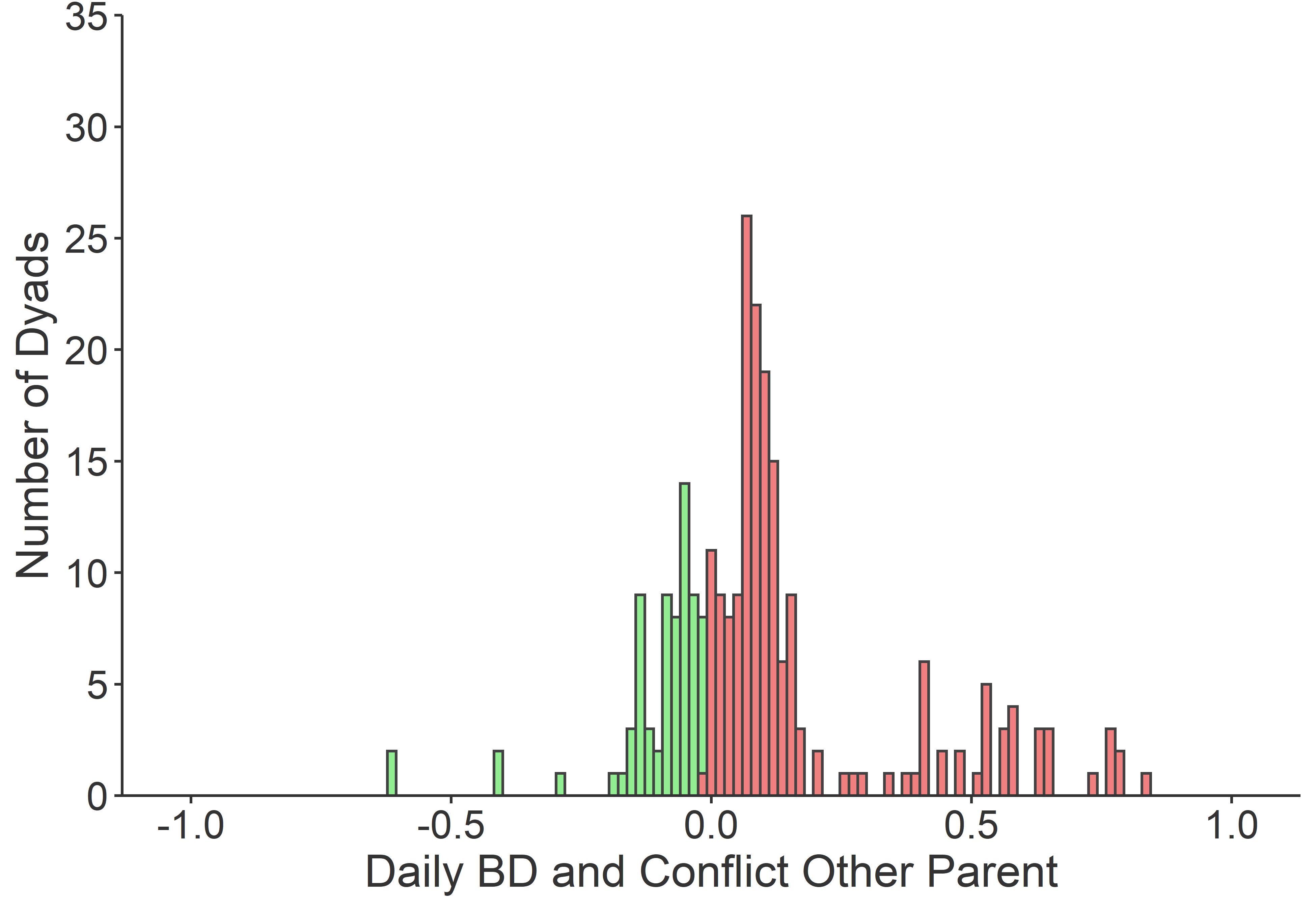

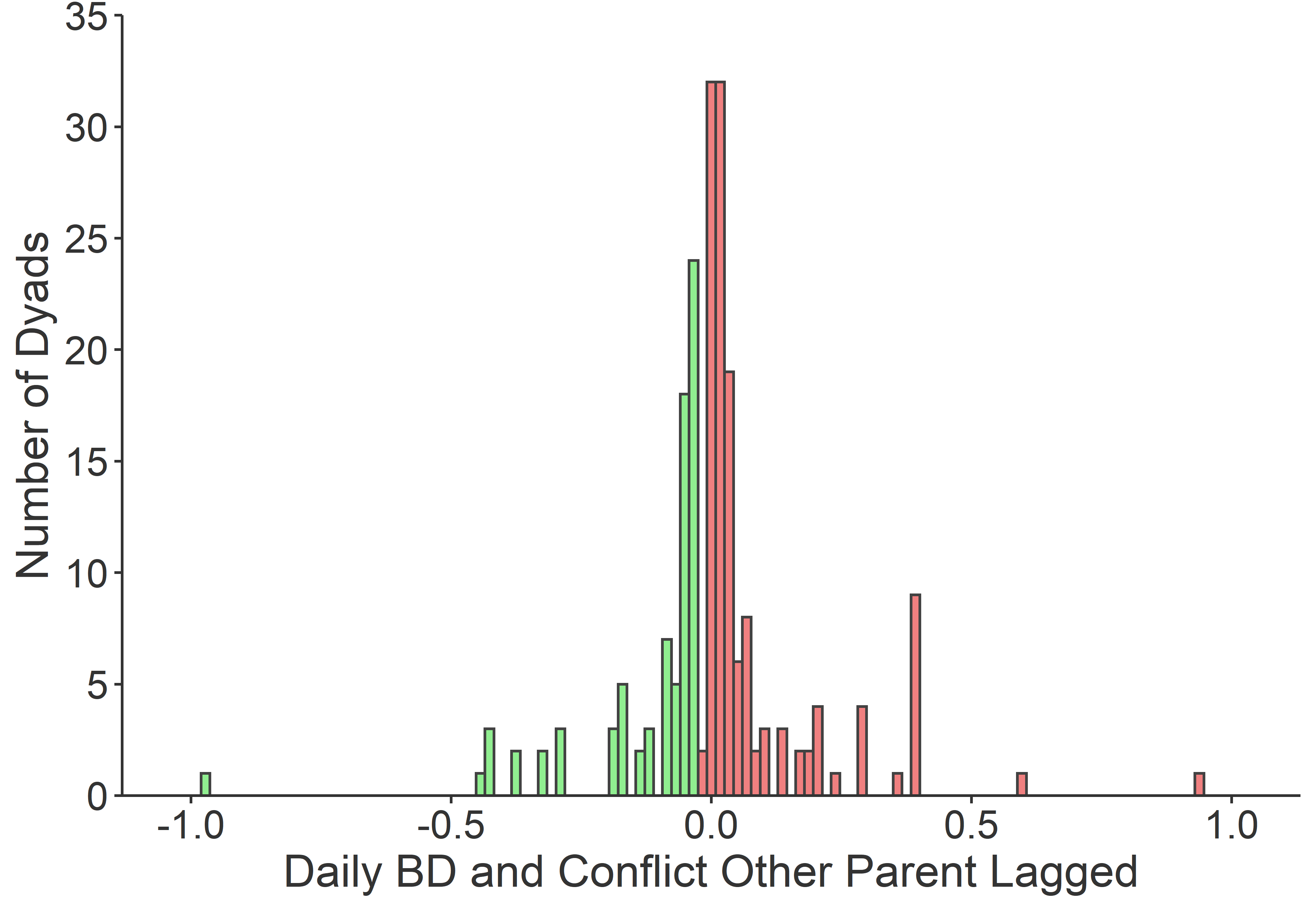

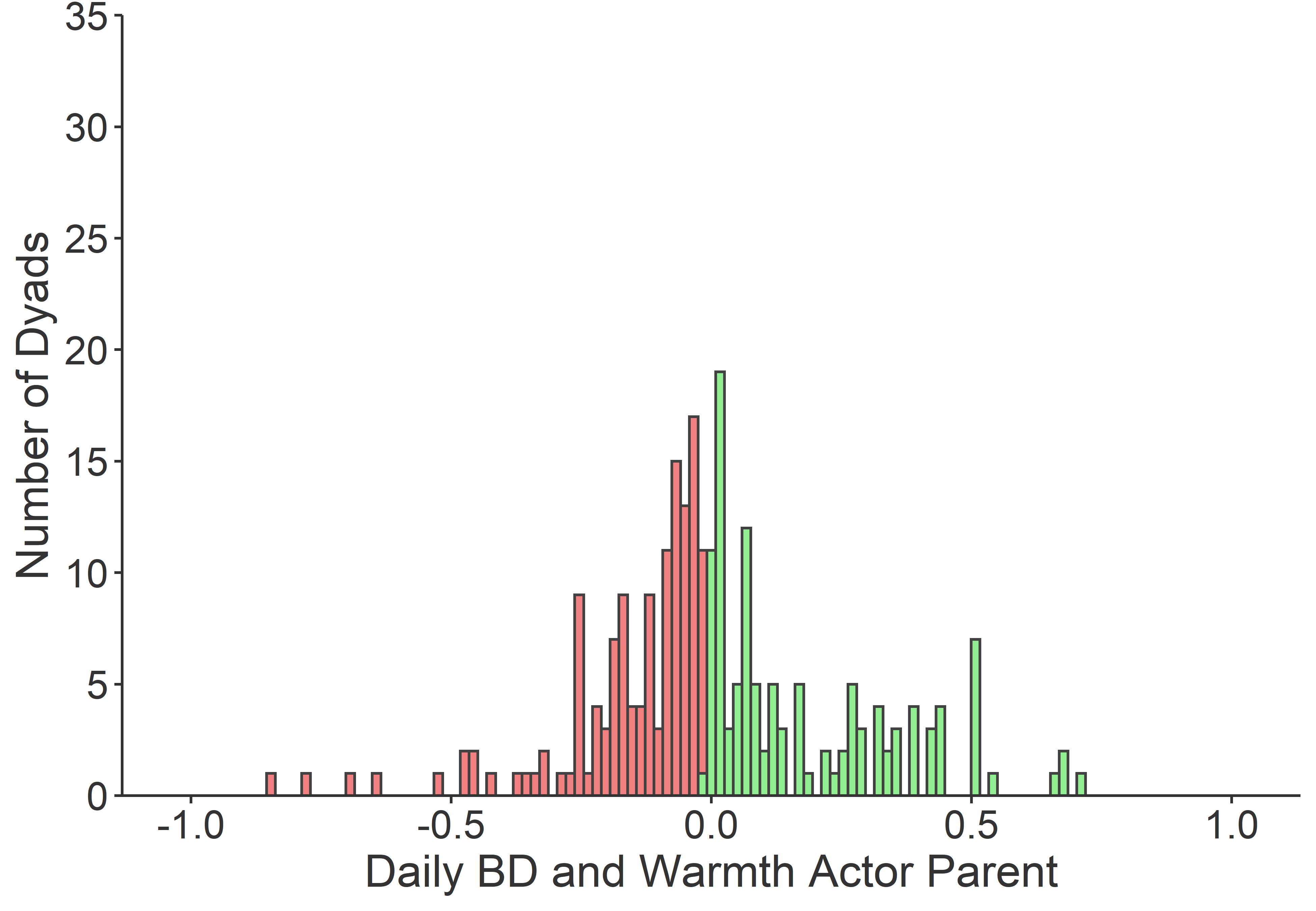

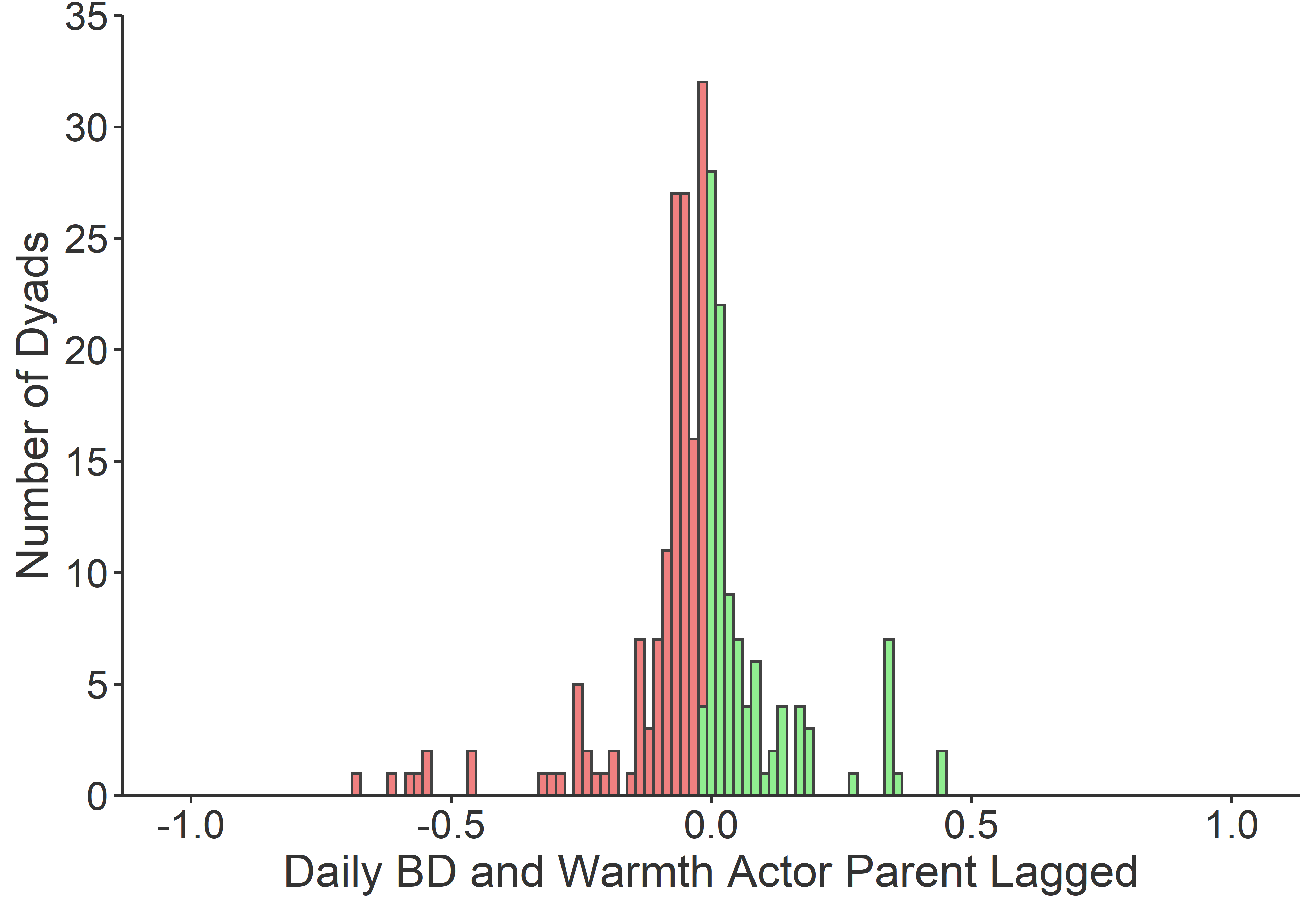

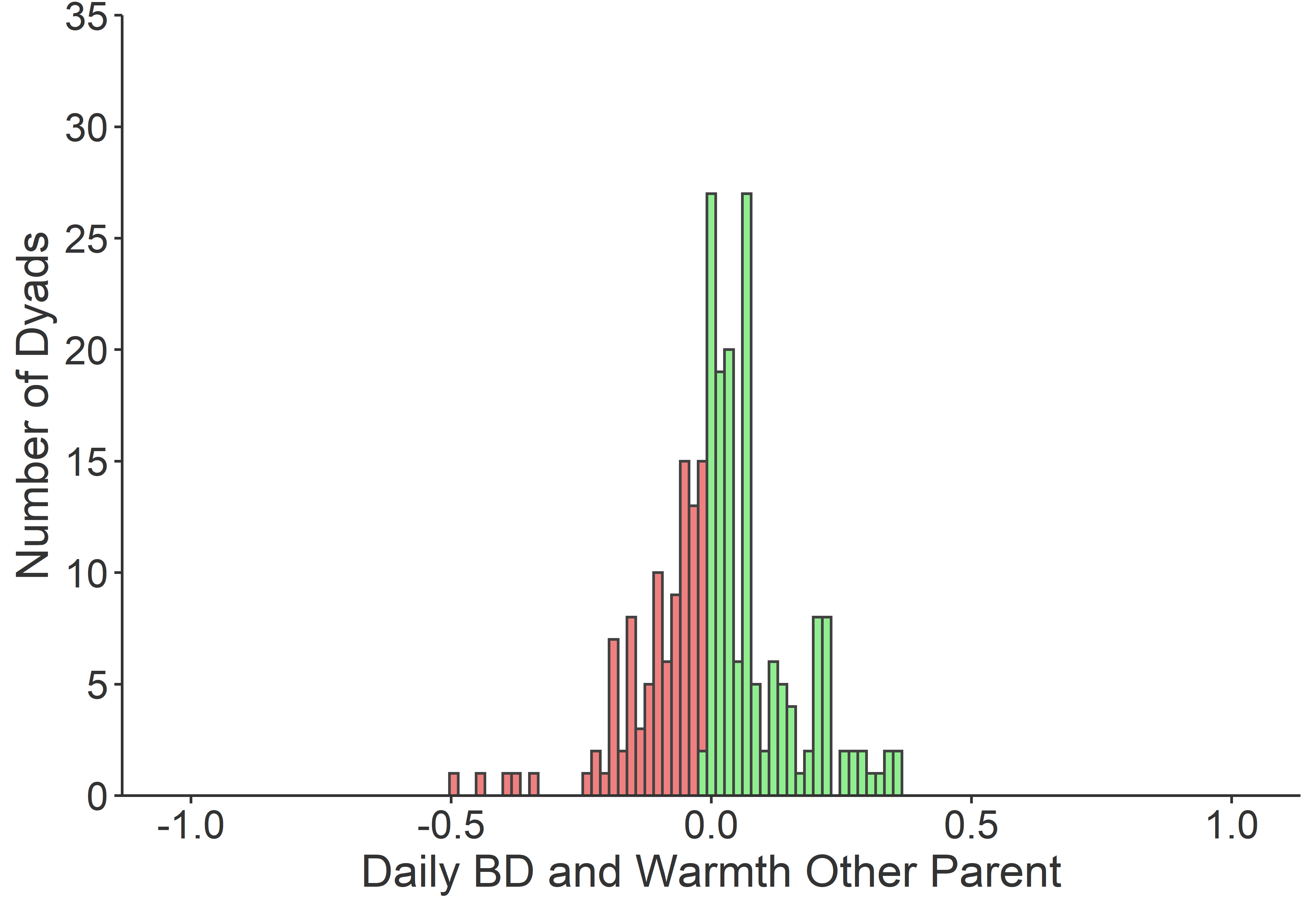

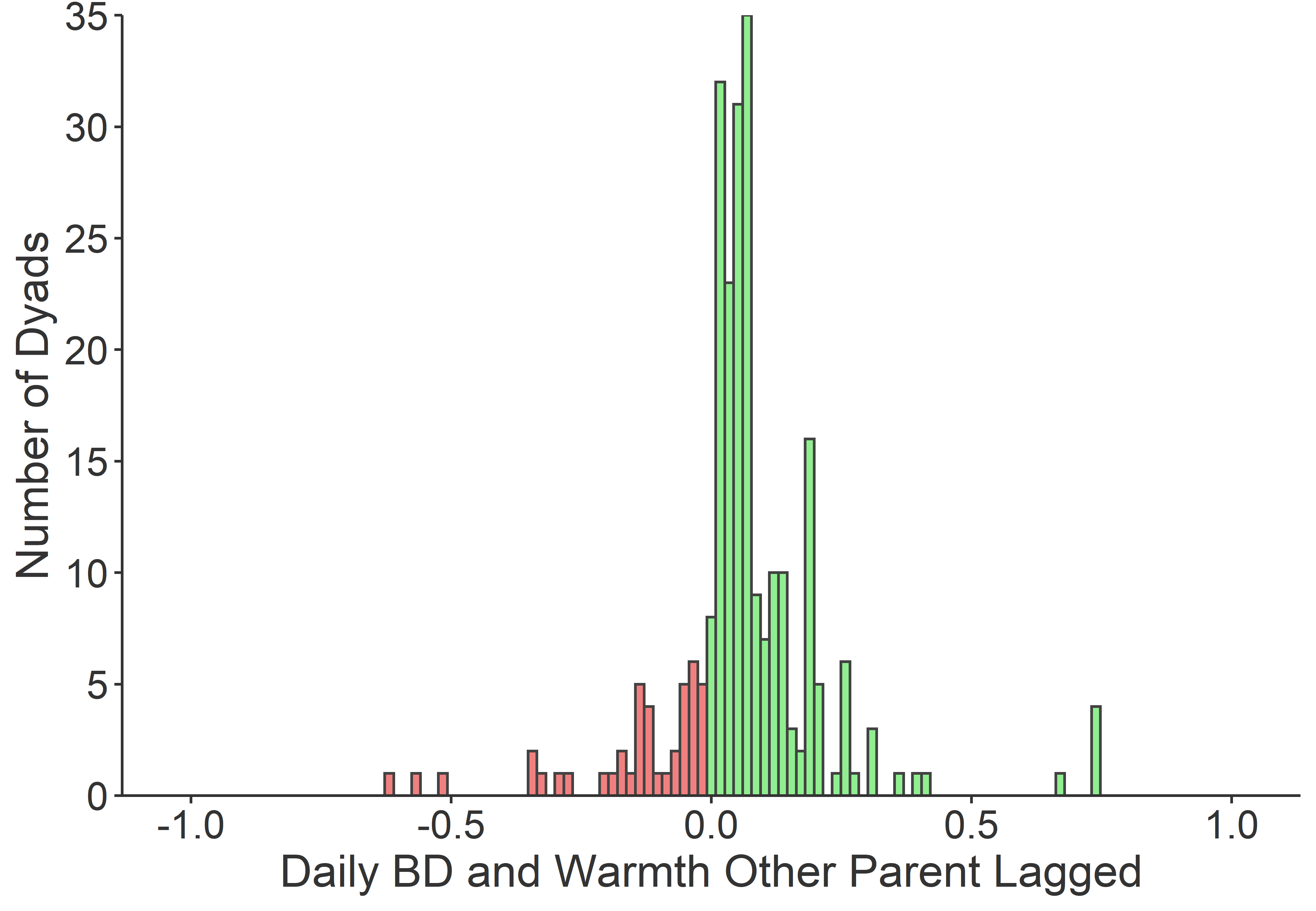


*Figures S10-S17.* Dyad’s half-yearly slopes (i.e., combining unique family slopes and unique dyad slopes). Red refers to worse and green to better relationship quality.


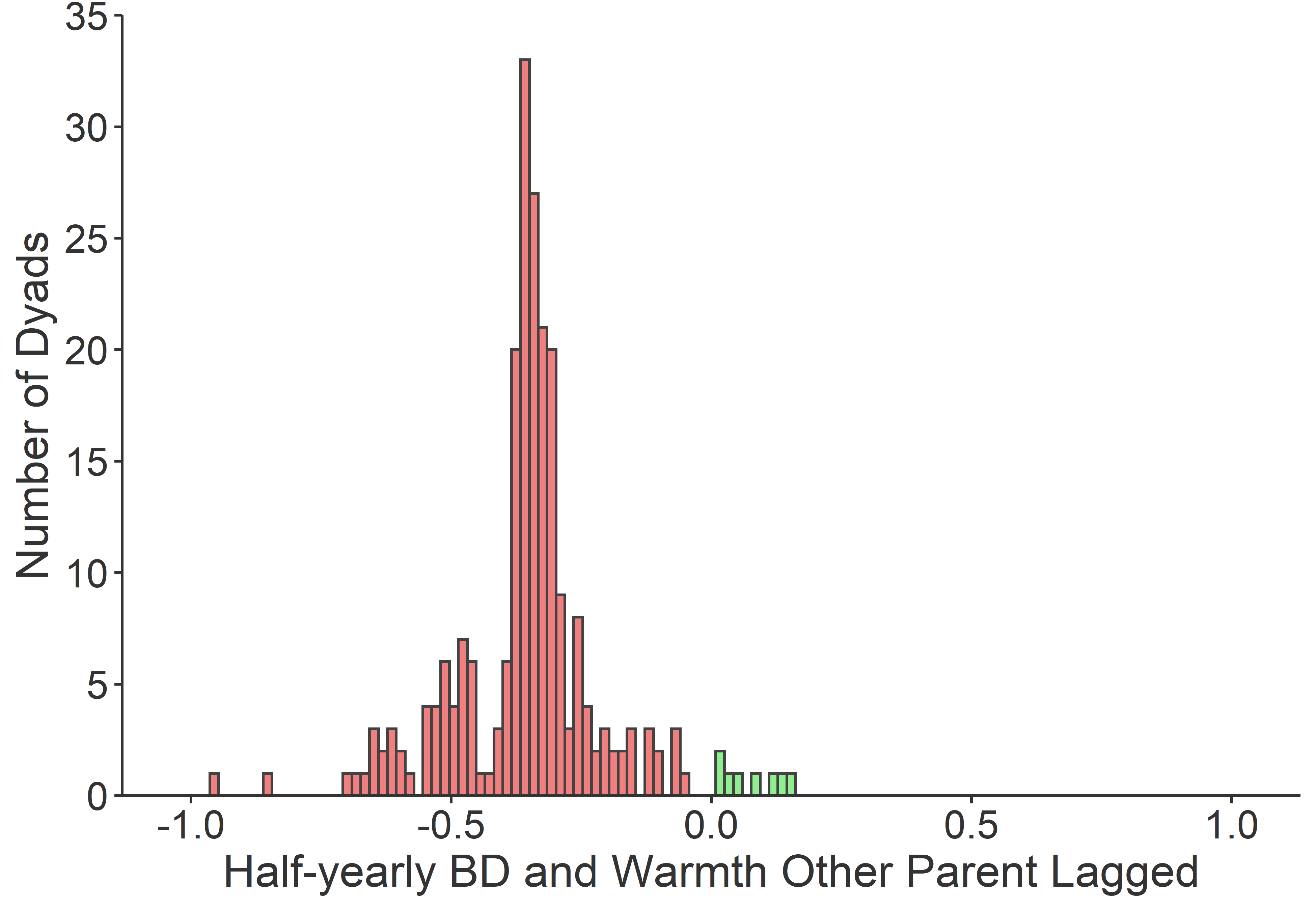

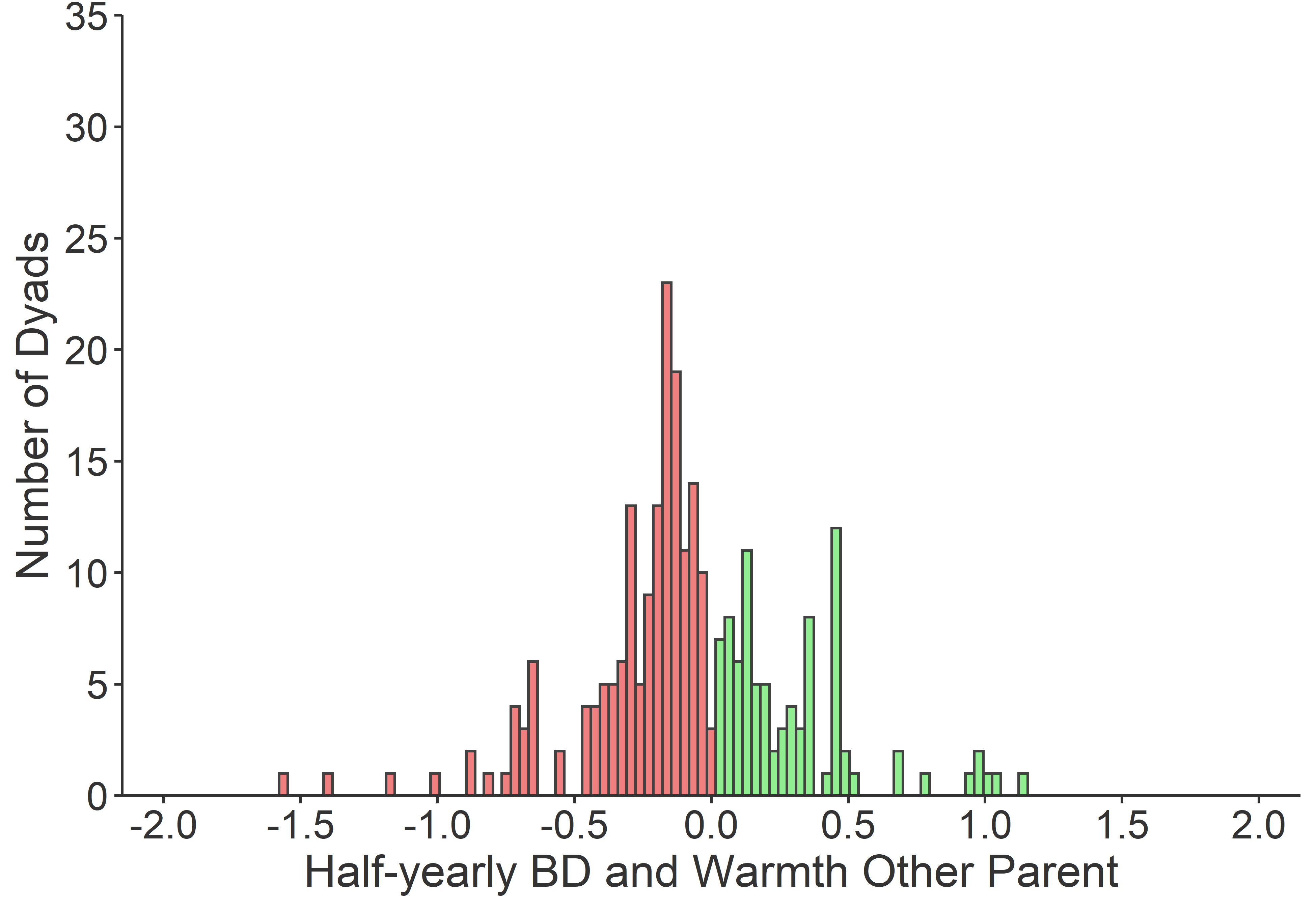

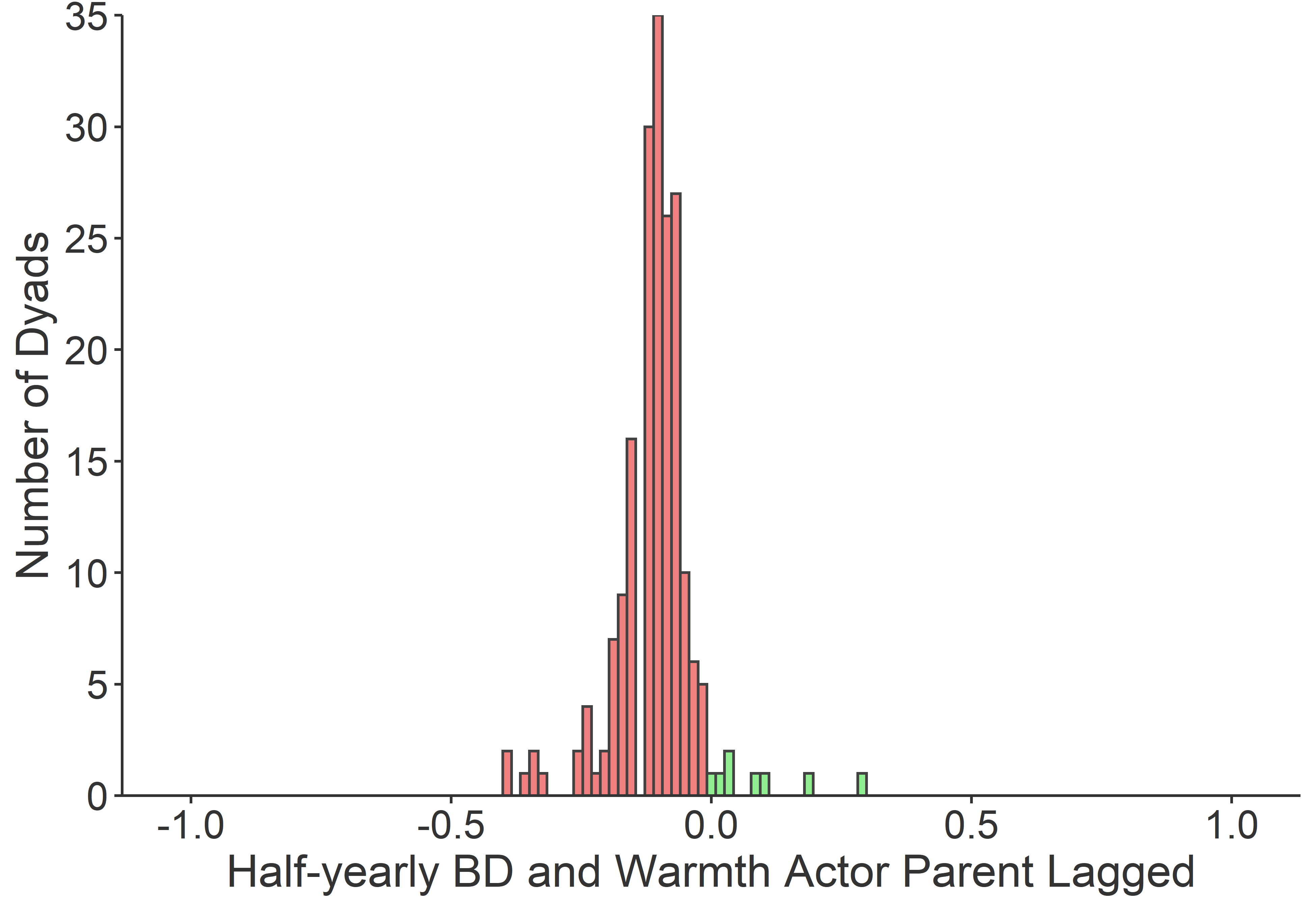

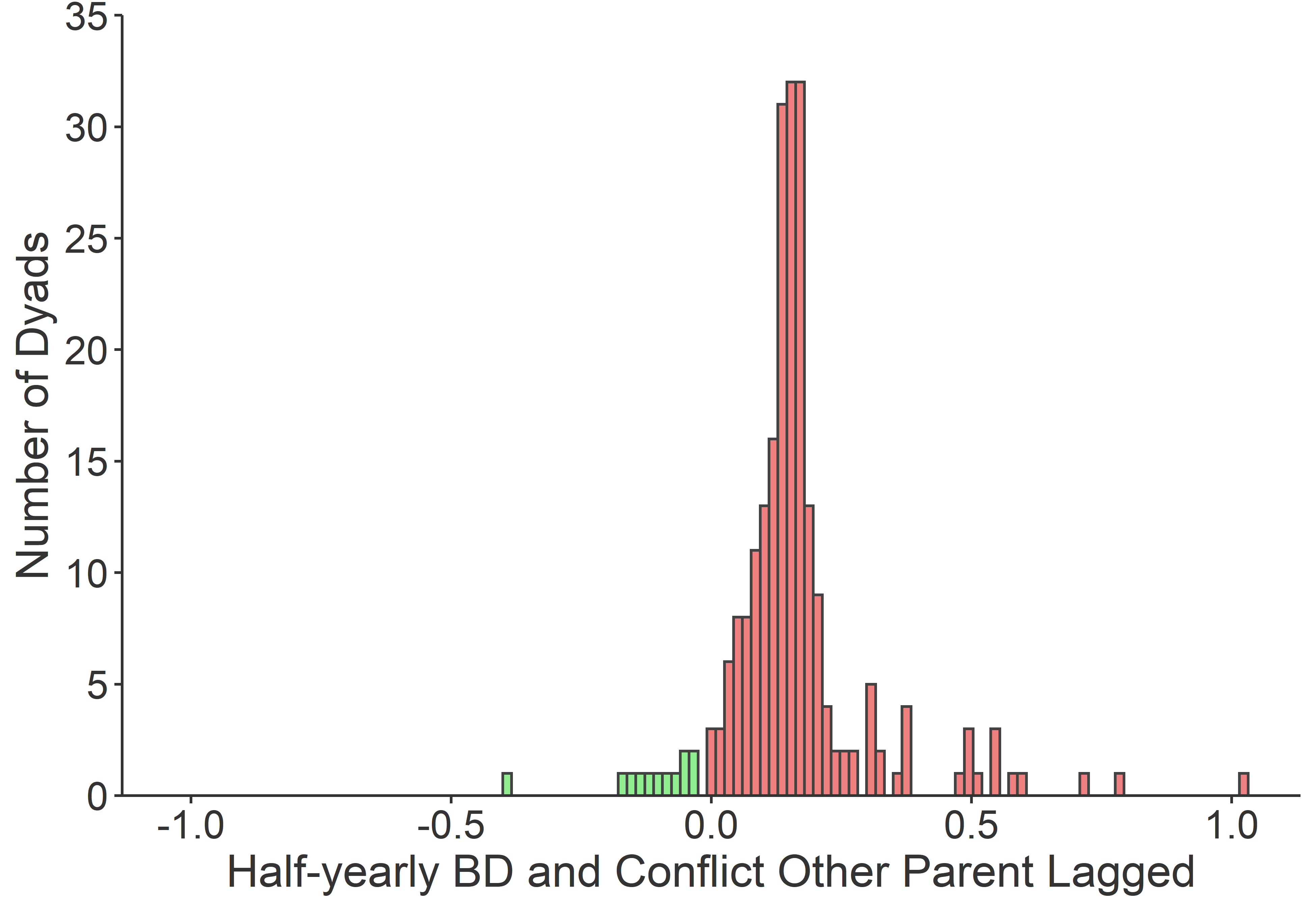

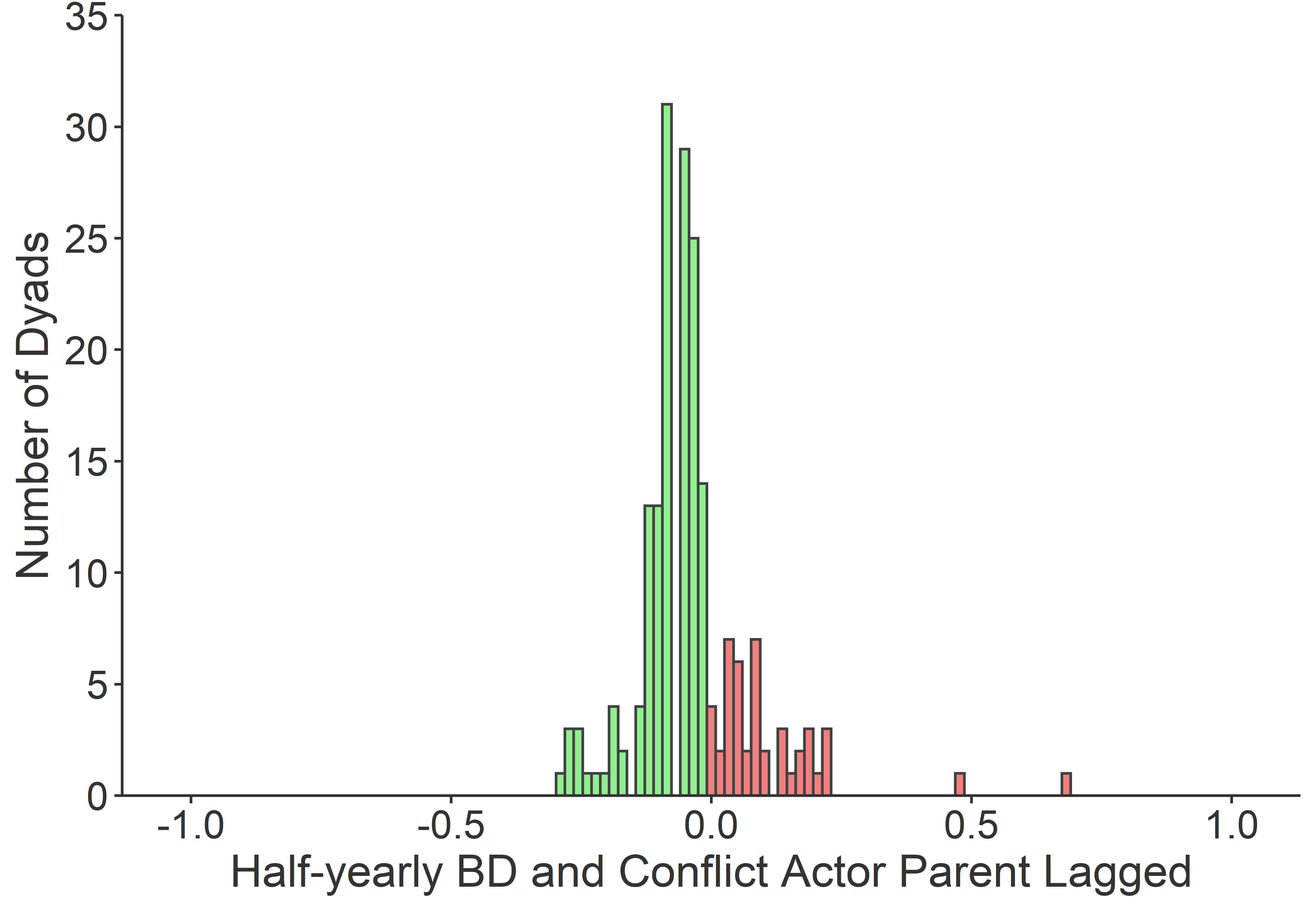

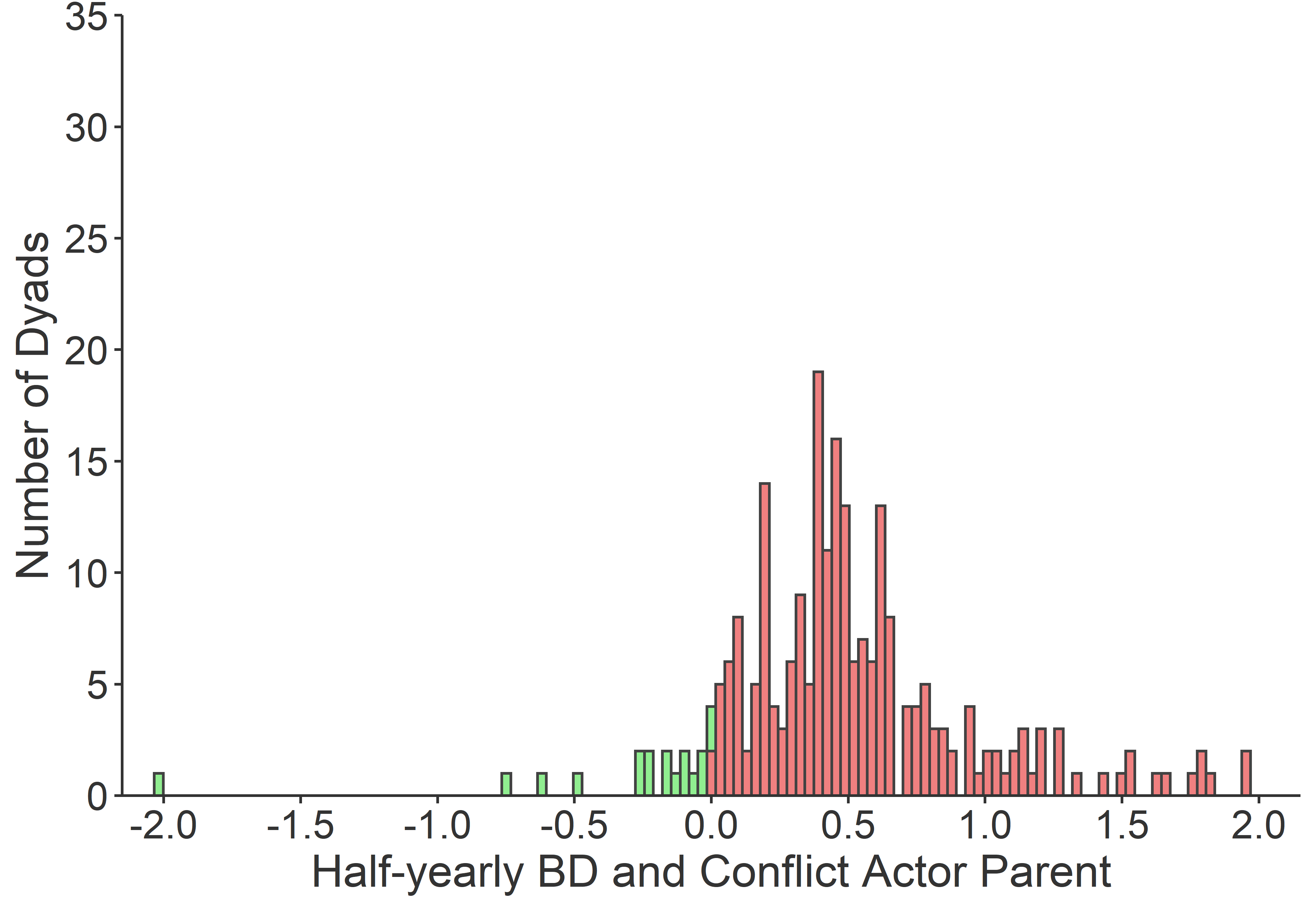

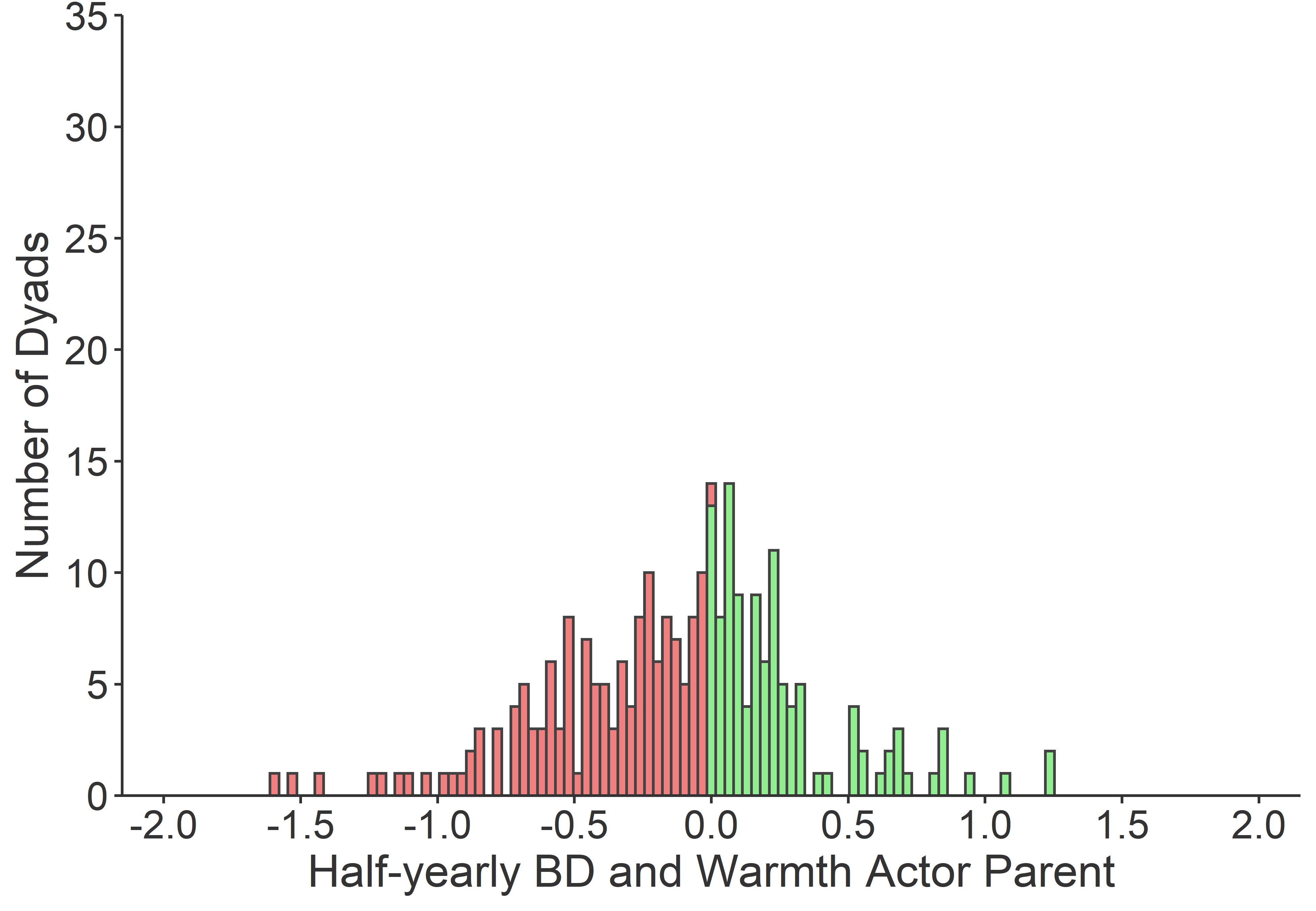

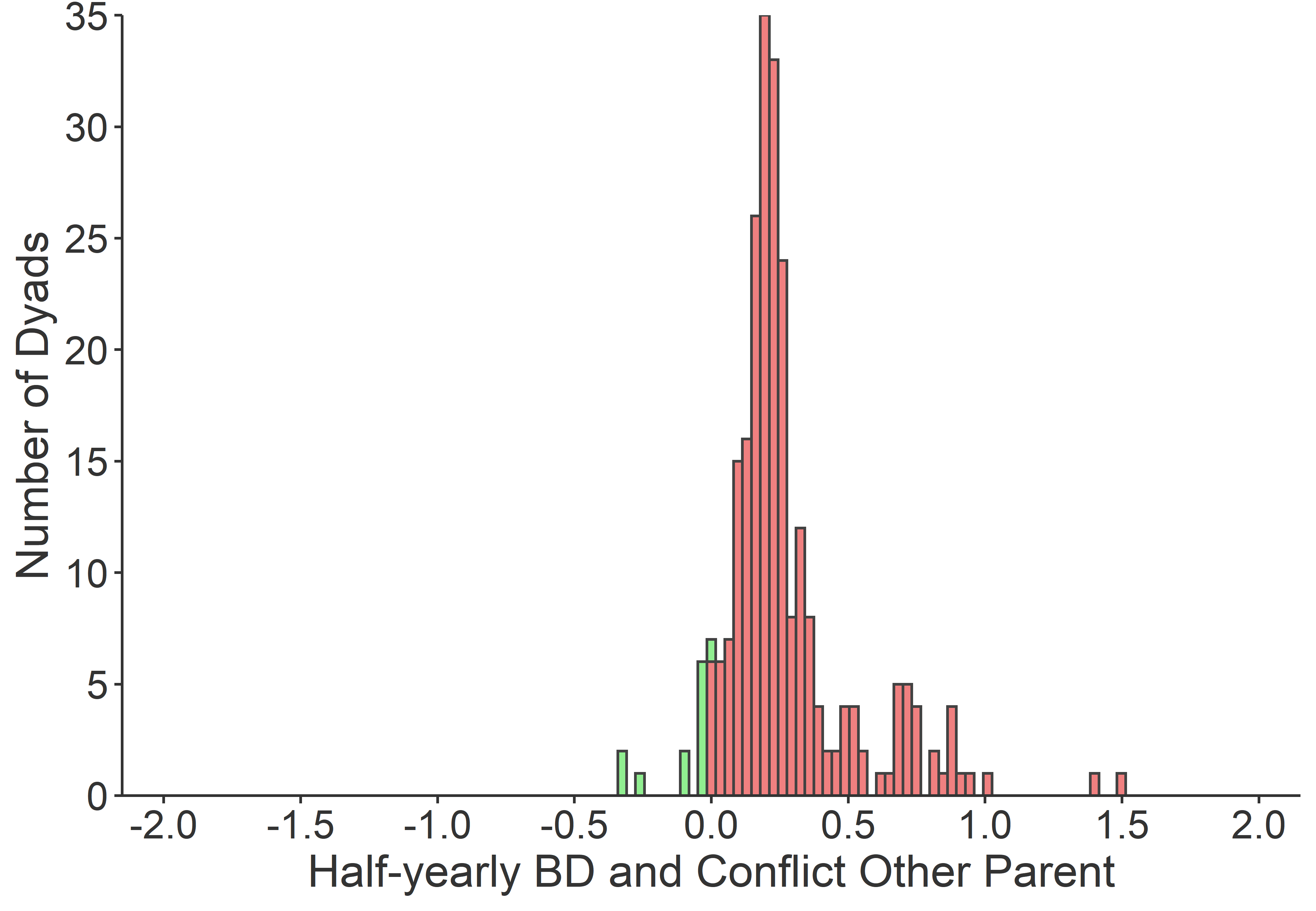

Supplement: Supplementary file 1 — Supplementary Information [file 10964_2024_2064_MOESM1_ESM.docx]
